# Land use change, carbon stocks and tree species diversity in green spaces of a secondary city in Myanmar, Pyin Oo Lwin

**DOI:** 10.1371/journal.pone.0225331

**Published:** 2019-11-26

**Authors:** Helen, Marcin Pawel Jarzebski, Alexandros Gasparatos

**Affiliations:** 1 Graduate Program in Sustainability Science-Global Leadership Initiative, Graduate School of Frontier Sciences, the University of Tokyo, Kashiwa City, Japan; 2 Institute for Future Initiatives, the University of Tokyo, Tokyo, Japan; University of Wisconsin Milwaukee, UNITED STATES

## Abstract

Myanmar undergoes rapid urban expansion and experiences its negative impacts, often due to the loss of urban green spaces. National and local authorities lack sufficient knowledge, capacity and plans on how to preserve urban green spaces and benefit from their ecosystem services, with such gaps being particularly pronounced in the smaller secondary cities. This study focuses in such as secondary city, Pyin Oo Lwin, and analyzes land use and land cover (LULC) change, tree diversity and carbon stored in aboveground and belowground biomass, and soil. We focus on the main green spaces of the city, which contain different configurations of urban forest, grassland and agricultural land. Remote sensing analysis tracked LULC change between 1988 and 2018, and showed the extensive increase of built-up area, and the decline of urban forests and urban farms. Even though a substantial amount of green spaces has been converted to built-up land, the remaining urban green spaces are still serving as an important habitat for many different tree species, with a total of 82 species from 35 families observed in the different green spaces. Furthermore, these green spaces contain significant carbon stocks, which are, however, highly variable: botanical garden (383.67 t/ha), coffee farms (355.64 t/ha), monasteries (277.14 t/ha), golf course (208.45 t/ha), and seasonal farms (123.22 t/ha). Nevertheless, the extensive LULC change has reduced carbon stocks from 2.41 Mt (1988) to 1.65 Mt (2018). The findings of this study provide a better understanding of LULC change in secondary cities of Myanmar, and build an evidence base on how urban green spaces preservation and green infrastructure development can contribute to green economic transitions, and sustainable, resilient, and low-carbon cities in the country.

## Introduction

The global urban population has increased rapidly from 751 million people in 1950, to 4.2 billion in 2018, with Asia currently accounting for 54% of this global urban population [1]. In Southeast Asia, 47% of its 294 million people live in urban areas, with this fraction expected to increase to 65% by 2050 [2].

Myanmar, as many other countries in the region, has been undergoing an urbanization transition. In some respect this urbanization transition is in its early stages but shows some interesting patterns. For example, Myanmar’s urbanization rate is relatively average for regional standards standing at 0.21–0.48%, when the urbanization rates of Malaysia, Bhutan, Indonesia, Bangladesh, Vietnam, and East Timor can be characterized as high (0.48–1.00%); and of Laos and Thailand as ultra-high (>1.00%) [3]. However, urban population density has increased rapidly from 6,200 people/km^2^ in 2000, to 7,500 people/km^2^ in 2010 [4]. Currently, approximately 30% of the total national population (51.48 million) lives in urban areas (increasing from 24.8% in 1983) [5]. This urban population is projected increase to 32.9% in 2025 and 34.7% in 2030 [6].

The gradual shift in political, social, and economic realities has been a major driver of this urban transformation in the country (and vice versa) and has been exemplified by a dynamic rural-urban migration. This is expected to affect profoundly Myanmar’s demographic, socioeconomic and environmental situation, but urban policy formulation is merely in an embryonic stage and not in a position to anticipate the sustainability challenges that urbanization will bring [7,8]. So far, due to the lack of a coherent urban policy framework in Myanmar [9], urban expansion has occurred in many cities without proper planning and management. This has resulted in an extensive and largely uncontrolled land use and land cover (LULC) change as witnessed by an ongoing rapid building development, loss of green spaces, and major traffic congestion in some of its major cities [10–14]. Regarding the latter, the recent relaxation in vehicle import regulations has resulted in a noticeable influx of vehicles [15], with the transport sector being responsible for 28% of the national CO_2_ emissions [16]. It is projected that the growing urbanization and increasing demand for urban transportation services will lead to a dramatic increase in greenhouse gases (GHG) emissions [17]. At the same time, many cities in Myanmar are exposed to the hazards posed by climate change such as intense rainfall, prolonged droughts, and unpredictable tropical storms, and heatwaves [18].

Such urban challenges have prompted city authorities and decision-makers in Myanmar to steer urban development towards a sustainable, climate resilient, and low-carbon future through the development of proper urban plans and policy frameworks [16,19,20]. At the same time, the national government has embarked in an effort to transition to a green economy [21,22]. Various ongoing consultations, negotiations and policy formulations between various stakeholders aims to identify possible pathways to achieve this transition [23,24]. Investing in natural capital and sustainable cities are two of the three key priority areas in enabling green economic transitions in Myanmar [25]. However, most of the above initiatives have focused on the major cities such as Yangon, Mandalay and Nay Pyi Taw [17], with little initiative and capacity to deal with such challenges in the many secondary cities [8].

The conservation of urban ecosystems and the development of green spaces/infrastructure are major interventions for improving the quality of urban life [26] and facilitating green economic transitions [27,28]. This is because urban ecosystems and urban green spaces such as parks, urban forests, allotments, street trees, green roofs, and urban farms, among others [29], provide numerous ecosystem services that can enhance the quality of life in cities and to contribute to climate change mitigation and adaptation [26].

Climate regulation is one of the major regulating services provided by urban green spaces [26,30]. Vegetation in urban green spaces sequesters and stores carbon by converting CO_2_ into aboveground and belowground biomass in stems, branches, leaves, and roots through photosynthesis [31,32]. Soils in urban green spaces also store substantial amounts of soil organic carbon (SOC) through biomass decomposition [33]. It has been suggested urban green spaces can serve as important carbon pools [34], thus offering a possible climate change mitigation option alongside all the other regulating and cultural services they provide [35,36]. At the same time urban green spaces are also important habitats for biodiversity, offering thus important supporting ecosystem services [37]. Many studies have highlighted the important role that urban green spaces play for carbon storage [33,38–43] and habitat for tree species [44–50]. However these outcomes can depend between cities and green spaces [41–53], with significant knowledge gaps remaining for some parts of the world and types of green spaces [51,52].

Harnessing the multiple benefits of urban green spaces could contribute substantially in the current efforts to achieve the interlinked goals of sustainable urban development and green economic transitions in Myanmar (see above). However, there are important knowledge gaps about urban land use change in Myanmar [53,54], with few studies about the benefits of urban green spaces [55,56]. On a regional scale, there are few studies on urban land use change, which mainly focus on major cities [57–59] and/or emphasize on single types of urban green space such as urban forests and trees [60]; [61] [62].

Considering the above, the aim of this research is to examine the interplay between urbanization and green spaces in a secondary city of Myanmar, Pyin Oo Lwin. In particular we explore how urbanization has driven the conversion of urban green spaces in past 30 years, and how the remaining green spaces serve as an important habitat for tree species and contain carbon stocks. We first track the LULC change effects of urbanization processes in Pyin Oo Lwin between 1988 and 2018. Second, we assess tree species diversity and carbon stocks in the main urban green spaces of the city, namely the botanical gardens, a golf course, monasteries, and urban farms used for coffee and food crop production.

The “Materials and methods” section outlines the methodology used to assess LULC change, tree species diversity and carbon storage in the different urban green spaces. We then outline LULC change patterns during the last three decades (1988–2018) (see “Land use and land cover change”), tree species diversity (see “Plant species diversity”) and carbon stocks (see “Carbon stocks”) in the different urban green spaces. The “Discussion” section revisits some of the main findings and discusses the possible benefits that the conservation of existing and the creation of new green spaces could have for meeting some of Myanmar’s policy objectives related to sustainable urban development and the transition to a green economy.

## Materials and methods

### Study site

**Fig 1:**
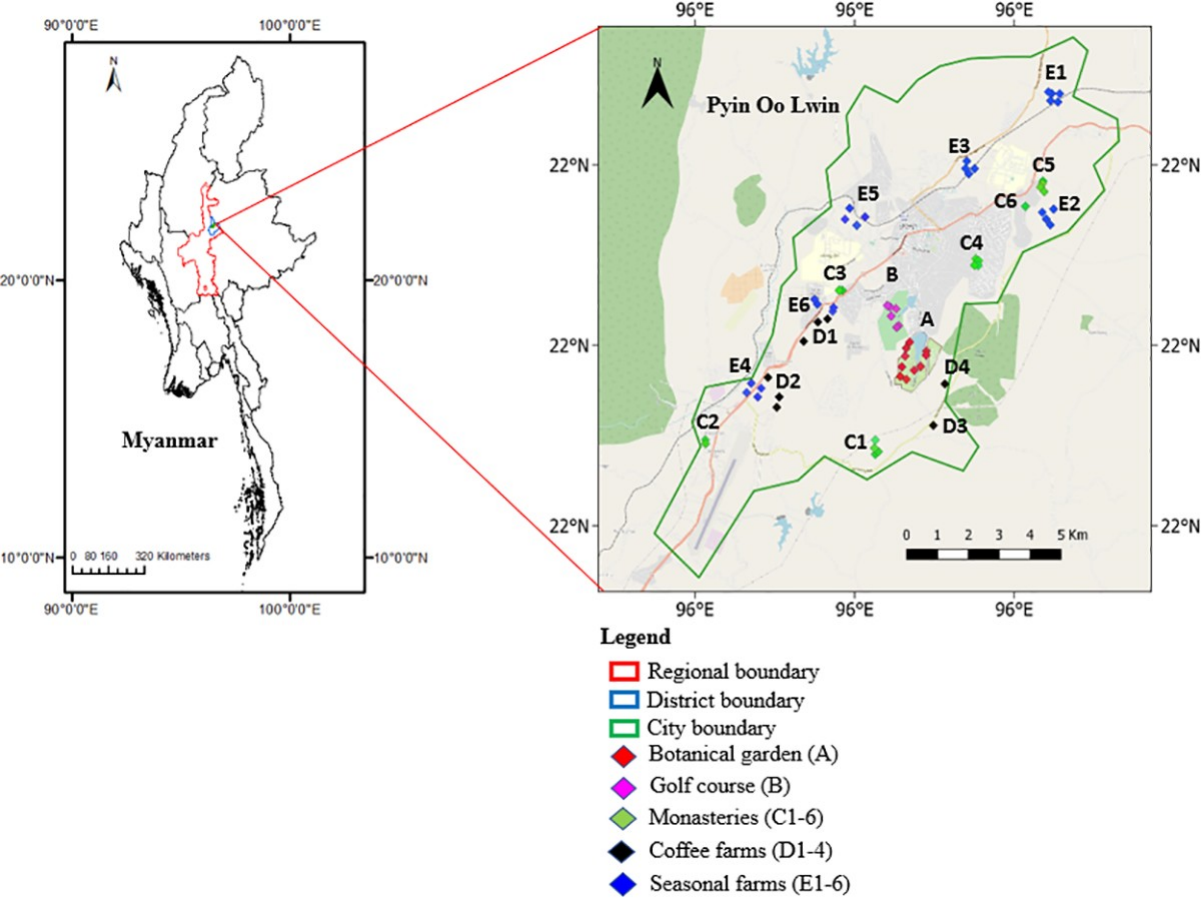
Location of Pyin Oo Lwin (left), green spaces and aboveground biomass surveys and soil sampling sites (right).

Pyin Oo Lwin is a secondary city in Myanmar. It is in the Shan Highlands, which is 67 km east of Mandalay with an altitude of approximately 1,070 m (Fig 1). The city was founded as a summer capital by the British in 1896. Currently, it is a city municipal area of approximately 102 km^2^, consisting of 21 wards with an urban population of 158,783 people [63]. Similar to other Myanmar cities, Pyin Oo Lwin’s population and built-up land has been constantly increasing in the last decades (see section: Land use and land cover change).

Due to its pleasant climate and environment, and accessibility to natural scenic landscapes around the city, Pyin Oo Lwin is a major resettlement and tourism destination within Myanmar. Furthermore, the city was granted the 2^nd^ ASEAN Environmentally Sustainable Cities Award in 2011 for its air quality [64], and is currently under the green city pilot project of the Myanmar Government [65]. Partly due to these good environmental conditions, real estate demand is steadily increasing, and built-up areas are gradually expanding to make space for new residents.

As other cities, the Pyin Oo Lwin urban area still contains natural resources such as forest areas, fertile land for agriculture, and water resources, while numerous urban green spaces play an important role in city life. The city hosts one of the most well-known urban green spaces in Myanmar, the National Botanical gardens (Kandawgyi Park), established in 1915 after the Royal Botanical Gardens Kew in England. The city golf course is another major green space established in 1896 at the same time as the development of the city. A unique cultural aspect of Pyin Oo Lwin is that the many scattered Buddhist monasteries have preserving pockets of urban forests. Furthermore, the city contains many urban agricultural areas, including many small family farms (mainly for food crops, vegetables and flowers), and larger coffee farms (both private- and government-owned). Coffee farming was introduced by the Roman Catholic missionaries in 1930 [66], with Pyin Oo Lwin becoming known as the coffee capital of Myanmar.

For this research we focus on the four main types of urban green spaces in the city namely, an urban park (Pyin Oo Lwin Botanical Gardens), a golf course, six monasteries, four coffee farms, and 25 seasonal farms. These are a good representation of the green spaces currently existing in the city, namely urban forest, grassland, and urban agricultural land. When selecting green space categories we used the green space interpretations suggested in the literature, which includes urban farms [67]. The actual green spaces were identified and selected through extended visits in the city and after consultation with local city officials from the planning department and the municipal office of the city.

In particular, the urban park (A, Fig 1, Table 1) and the golf course (B, Fig 1, Table 1) are unique green spaces within the city. There is a lack of other extensive parks within the city, with most public recreational spaces containing only sparse vegetation. In fact, most of the remaining urban forest is located within monasteries and military bases. The six selected monasteries (C1-C6, Fig 1, Table 1) represent a good spread of sizes and locations within the city. Unfortunately, despite repeated requests it was impossible to gain access to military bases. Regarding urban agricultural areas, we selected two private and two government coffee farms within the city boundaries (D1-4, Fig 1, Table 1). For seasonal farms, we identified the main farm clusters within city boundaries through discussions with the city municipality office and a key informant (the main agricultural supply store owner). Farms in individual clusters (E1-6, Fig 1, Table 1) were randomly selected through transect walks and subject to the availability of the farmers, and their willingness to provide access to their property.

**Table 1:** Characteristic of the study green spaces.

Code	Area (ha)	Uses	Main vegetation
A	177.00	Botanical garden (Kandawgyi)	Urban forest, grass land
B	104.59	City golf course	Urban forest, grass land
C1	20.82	Nyaung Ni Monastery	Urban forest
C2	4.27	Nwe Hninn Monastery	Urban forest
C3	1.33	Kyaut Taung Monastery	Urban forest
C4	5.68	Shwe Si Khone Monastery	Urban forest
C5	7.14	Migadarwon Monastery	Urban forest
C6	1.83	Aye Chan Thar Yar Monastery	Urban forest
D1	132.05	Government-owned coffee farm	Coffee, shading trees
D2	86.30	Government-owned coffee farm	Coffee, shading trees
D3	23.30	Privately-owned coffee farm	Coffee, shading trees
D4	3.07	Privately-owned coffee farm	Coffee, shading trees
E1	1.25	Seasonal farm	Seasonal vegetables, flowers
E2	2.12	Seasonal farm	Seasonal vegetables, flowers
E3	0.57	Seasonal farm	Seasonal vegetables, flowers
E4	1.50	Seasonal farm	Seasonal vegetables, flowers
E5	1.42	Seasonal farm	Seasonal vegetables, flowers
E6	0.97	Seasonal farm	Seasonal vegetables, flowers

All necessary permissions for fieldwork were received through applications to the Ministry of Natural Resources and Environmental Conservation, and the relevant local authorities at Pyin Oo Lwin. Access to the individual green spaces was achieved after discussions with the management of the Botanical Gardens and the golf course, the abbots/monks of the different monasteries, the managers of coffee farms and individual family farmers. Through these discussions we stressed the academic nature of this study, its purpose and that no personal information will be collected.

### Data collection and analysis

Initially through remote sensing techniques we characterize land use patterns in the city during different time periods and estimate LULC change between 1988–2018 (Stage 1) (see section: Land use and land cover (LULC) change analysis (Stage 1)). We then undertake biomass surveys and soil sampling in 66 plots spread across the main land use classes and types of green spaces encountered in the city (Stage 2) (see section: Vegetation and soil sampling (Stage 2)). Subsequently we estimate the abundance and diversity tree species (Stage 3) (see section: Tree species diversity (Stage 3)) and carbon storage capacity using allometric equations (for aboveground/belowground biomass) and soil analysis (for SOC) (Stage 4) (see section: Carbon stock estimation (Stage 4)). The training samples for land use classification, biomass surveys, soil samples, and tree species diversity surveys were conducted in February-March 2018.

#### Land use and land cover (LULC) change analysis (Stage 1)

To estimate the extent of urban green spaces within the city, and their change over time we conduct a LULC analysis for the years 1988, 1998, 2008, and 2018. These specific years were selected as they mark some important national policy milestones, which have influenced urban expansion in Myanmar. In particular, in 1988 the national government initiated market-oriented reforms, which acted as a major driver of urbanization [68] (see section: Introduction). In 2008 there was a major shift in urban governance, as the 2008 constitution law assigned the management of the housing sector including urban development of the States and Regions [17].

For the LULC analysis we used the open source software, Quantum Geographic Information System (QGIS), version 2.16.0. Cloud-free multispectral Landsat images with the datum WGS-84 of the same period were collected from the earth explorer USGS web portal. The satellite pictures covered the entire boundary of Pyin Oo Lwin as identified through administrative maps provided by the municipal planning department. The collected satellite images (see S1 Table) were analyzed through the algorithms: pre-processing, image classification, accuracy assessment, and land use transition matrix.

Initially, a pre-processing, which involves geometric and radiometric corrections, was performed using the Semi-automatic Classification Plugin (SCP). Since L1TP imagery products involve the standard terrain correction [69], only the radiometric correction was carried out in this study. The radiometric correction was employed through the dark object subtraction (DOS) [70], which nullifies the haze component caused by additive scattering from remote sensing data [71,72]. Then we generated through SCP a Landsat virtual raster that is a composite image of Landsat’s full spectral bands, with exception for thermal bands. Afterwards, the virtual raster was converted into a false color composite image by using the RGB ratio 4-3-2 to distinguish different land uses and vegetation categories.

After pre-processing, the false color virtual raster was classified by using the supervised maximum likelihood classification technique [73]. In this classification, the region of interest (ROI) was created manually for each feature and training samples of signature fields were produced by means of polygon vectors. Afterwards, the maximum likelihood classifier (MLC) [74] was performed to obtain the classified image. In this study, seven land use classes were identified from the satellite imagery, namely: water, build up land, urban forest, urban agricultural area (seasonal farms), urban agricultural area (coffee farms), grass land, and other land use. S2 Table in the Supporting information provides more explanation about the characteristics of each land use type.

After image classification, we performed accuracy assessment and land use change detection through functions under the postprocessing tab in SCP. To validate the classified image, 100 random sampling points were used for each year [75]. Land use classes were determined through Google Earth images for the different assessment years. To improve the accuracy, we have verified the classified images with the false color images, the NDVI values from the composite images, and with the ground checkpoints collected during the field survey in February-March 2018. Subsequently, the overall accuracy and the kappa coefficient were computed. The producer's accuracy represents the error of omission (underestimation) and the user's accuracy refers to the error of commission (overestimation) [76]. The Kappa coefficient can be interpreted as follows: poor accuracy (<0.20), fair accuracy (0.20–0.40), moderate accuracy (0.40–0.60), good accuracy (0.60–0.80), and very good accuracy (0.80–1.00) [77].

Finally, the LULC change was assessed through the comparison between the classified images of the selected dates. A cross-tabulation analysis was employed for 1988–1998, 1998–2008, 2008–2018, and 1988–2018, with land use transition matrices generated [78].

#### Vegetation and soil sampling (Stage 2)

Stratified random sampling was used to identify the sites of the biomass surveys and soil sampling in each green space (S1 Fig). For the biomass survey we followed the sampling design suggested by the UN Food and Agriculture Organization [79] (S2 Fig). Initially we established primary sample plots of 20×20 m, with nested plots of 10×10 m and 1×1 m inside the primary plot. Trees with DBH ≥ 5 cm (diameter at breast height) and H>1.3 m (height) were measured in the 20×20 m plot, while the trees with DBH<5 cm and H>1.3 m were measured in the 10×10 m plot. The tree DBH and height were measured by using a diameter tape and a Trupulse laser respectively. The species and local names were recorded for all measured trees. Litter, grass, shrubs, and herbs were collected from the 1×1 m plot. We recorded their weight in the field with appropriate scales, and were then oven dried to estimate the moisture content and calculate the dry weight for biomass estimation (see section: Carbon stock estimation).

A total of 41 20×20m sample plots were created between all green spaces. In particular 10 plots in the botanical garden (8 plots in the forested portion, and 2 plots in the grassland), 17 plots in urban forested areas of the different monasteries, 8 plots in coffee farms (6 plots from government-owned farms and 2 plots from private farms), and 6 plots in the golf course (3 plots in the forested portion, and 3 plots in the grassland). We did not conduct above and below ground biomass surveys in seasonal farms, given the general lack of trees (see section: Carbon stock estimation).

Soil samples were collected from the plots described above in the botanical garden, monasteries, coffee farms, and the golf course. In each of these plots five soil-sampling points were defined as indicated in S2 Fig. We also collected soil samples in 25 crop farms, with the number of soil samples in each farm varying with its size, following the survey design for home gardens [80] (see S3 Fig).

The soil samples from the designated sampling points were collected from 0–30 cm depth, using a soil auger. Then, the soils were mixed together in a bucket to obtain a composite sample for each plot. Approximately 1 kg of each soil sample was packed in a plastic bag for laboratory analysis to test soil organic matter (OM) and bulk density (BD). Overall, 66 soil samples were collected between all urban green spaces, including 10 samples from the botanical garden, 17 samples from the monasteries, 8 samples from the coffee farms, 25 samples from the seasonal farms, and 6 samples from the golf course.

#### Tree species diversity (Stage 3)

To examine tree species diversity we used the important values index (IVI) [81] and Shannon diversity index. The IVI comprises of three components (Eq 1):

IVI=Relativedensity+Relativefrequency+Relativedominance (1)

where density denotes the presence of individual species (Eq 1.1), frequency denotes the distribution of the species (Eq 1.2), and dominance denotes the abundance of species (Eq 1.3).

$$Density=\frac{No.of\;a\;species}{Total\;area\;sampled}$$ (1.1)

$$Frequency=\frac{Area\;of\;plots\;in\;which\;a\;species\;occurs}{Total\;area\;sampled}$$ (1.2)

$$Dominance=\frac{Total\;basal\;area\;of\;a\;species}{Total\;area\;sampled}$$ (1.3)

$$Relative\;density=\frac{Density\;of\;a\;species}{Total\;density\;of\;all\;species}\times 100$$ (1.4)

$$Relative\;frequency=\frac{Frequency\;of\;a\;species}{Total\;frequency\;of\;all\;species}\times 100$$ (1.5)

$$Relative\;dominance=\frac{Dominance\;of\;a\;species}{Total\;dominance\;of\;all\;species}\times 100$$ (1.6)

$$Basal\;area\;(BA)=\frac{\pi {\left(DBH\right)}^{2}}{40000}\;(m^{2})$$ (1.7)

The Shannon diversity index (Eq 2) [82] usually ranges between 1.5 and 3.5 [83], with higher values denoting higher species diversity.

$$Shannon\;diversity\;index\;(H^{\prime })=-\sum_{i=1}^{s}p_{i}lnp_{i}$$ (2)

where;

H' = index of species diversity

S = the number of species richness

*p_i_* = the proportion of individuals of each species belonging to the i^th^ species of the total number of individuals

#### Carbon stock estimation (Stage 4)

The aboveground biomass component was calculated using allometric equations obtained from the GlobAllomeTree website and other studies (S3 Table). To estimate more accurate carbon stocks, we selected allometric equations for the same species, same genus, and the same family from tropical region prioritizing equations from the Southeast Asia region. To estimate the aboveground biomass component of seasonal farms, following Zomer et al., (2016) we assume annual biomass production of 5 t/ha without any trees. Site visits suggested that trees were virtually absent in the seasonal farms. The belowground biomass component (e.g. for roots) was calculated using 0.24 as a default root to shoot ratio. The total carbon content of vegetation biomass (for both above and below ground components) was estimated using a carbon conversion factor of 0.47 [85].

The collected soil samples were sieved through a 2 mm mesh wire and were cleaned and air-dried for 48 hours until the weight was stable for analysis. The soil bulk density (g/cm^3^) was calculated as the proportion of the dry weight of soil (g) to the bulk volume of soil (cm^3^). To estimate the soil organic matter (OM) content, soil samples were analyzed using the loss on ignition (LOI) method, and a conversion factor of 0.58 between OM to soil organic carbon (SOC) [86,87]. Eq 3 was used to calculate SOC stock [88].

$$SOC\;stock\;(tonne{ha}^{-1})=SOC\;\left(\frac{g}{100g}\right)\times soil\;bulk\;density\;\left(\frac{g}{{cm}^{3}}\right)\times soil\;depth\;(cm)$$ (3)

Based on the above we calculate the total carbon stock of each LULC (i.e. urban forest, grassland, seasonal farms, coffee farms) as the sum of the aboveground, belowground, and SOC components (t/ha). We estimate average carbon stocks for each type of green space. For green spaces that contain only one LULC class (e.g. monasteries, coffee farms, seasonal farms), we average the scores of the different plots. For green spaces that contain a combination of LULC classes (e.g. botanical gardens, golf course), we calculated area-weighed scores for the carbon stocks. We then use these estimates to assess carbon stock change and density for 1988, 1998, 2008, and 2018.

We should note that as the underlying biomass and tree species surveys were conducted in 2018, the carbon stock estimates for 1988, 1998 and 2008 might be uncertain. This is because we do not know the exact tree species composition for the past years. In our analysis we assume that tree species composition in the past was the same as the 2018 composition for two reasons. First, according to the Forest Reference Level (FRL) of Myanmar the forest type in the study site is categorized as Hill Evergreen Forest. The tree species surveyed in each green space have strong analogies with Hill Evergreen Forest species. Similarly, coffee farms also tend to contain the same tree species over time, as they are long-term plantations in this study area. Second, we corroborated these observations through the review of secondary data and informal interviews with civil servants from relevant local government departments. This verification process has suggested that the forest type has not changed over time in the study region.

## Results

### Land use and land cover change

**Fig 2:**
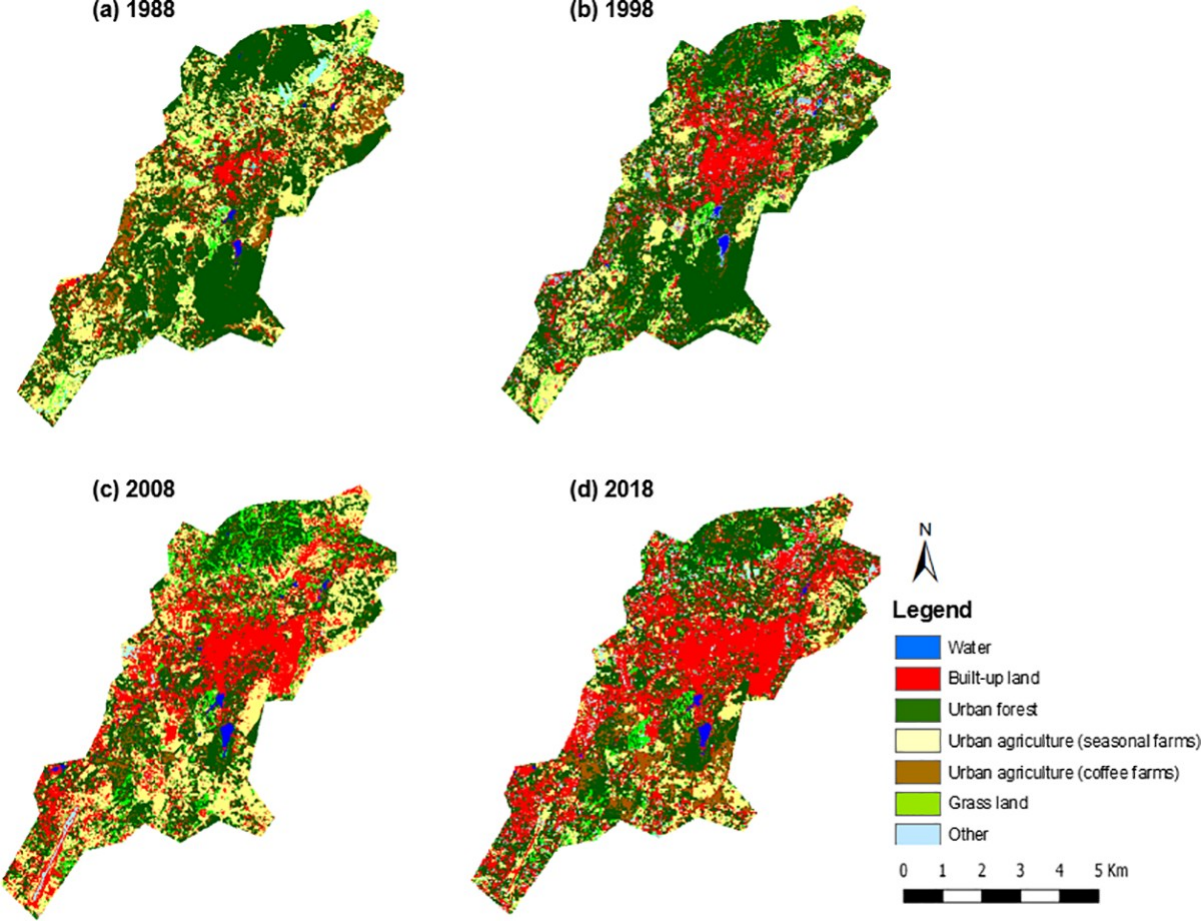
Land use maps of Pyin Oo Lwin city for 1988, 1998, 2008, and 2018.

Classified images were generated for the years 1988, 1998, 2008, and 2018 using seven urban land use classes (Fig 2). The overall accuracy of each classification is 89.11% (for 1988), 92.08% (for 1998), 85.15% (for 2008), and 97.03% (for 2018). Kappa coefficient were estimated at 0.85, 0.89, 0.81, and 0.96 for the years 1988, 1998, 2008, and 2018 respectively, suggesting very good accuracy [77]. The error matrix of each classification is summarized in S4 Table.

Table 2 contains the estimated extent of the different land cover classes. Results suggest the gradual expansion of built-up land and the decline in urban green spaces in the past three decades (1988–2018). In particular, the urban built-up land increased 5-fold, from 730.64 ha in 1988, to 1499.18 ha in 1998, 2055.85 ha in 2008, and 3607.20 ha in 2018. By 2018 built-up land constituted 35.50% of the Pyin Oo Lwin administrative area (up from 7.19% in 1988).

**Table 2:** LULC in 1988, 1998, 2008, and 2018 (ha).

	1988		1998		2008		2018	
Land cover	Area	%	Area	%	Area	%	Area	%
Water	29.58	0.29	37.04	0.36	37.21	0.37	48.94	0.48
Built-up land	730.64	7.19	1499.18	14.76	2055.85	20.24	3607.20	35.50
Urban forest	5337.71	52.54	5466.58	53.81	4901.63	48.25	3558.36	35.02
Urban agriculture (seasonal farms)	2966.53	29.20	1845.91	18.17	2057.94	20.26	1410.71	13.89
Urban agriculture (coffee farms)	779.57	7.67	446.59	4.40	411.96	4.05	756.58	7.45
Grass land	180.78	1.78	426.16	4.19	352.27	3.47	383.73	3.78
Other	135.02	1.33	438.37	4.31	342.96	3.38	394.30	3.88
**Total**	10159.82	100.00	10159.82	100.00	10159.82	100.00	10159.82	100.00

At the same time, we identify a significant decrease in urban forest area between 2018 (3558.36 ha) and 2008 (4901.63 ha), with relatively lower variation between 1988 (5337.71 ha) and 1998 (5466.58 ha). In 2018, the urban forest constituted only 35.02% of the Pyin Oo Lwin administrative area, compared to 52.54% in 1988. There was a fluctuation in the area under seasonal farms areas over time: 1988 (2966.53 ha), 1998 (1845.91 ha), 2008 (2057.94 ha), and 2018 (1410.71 ha), which still suggests a long-term decrease. Coffee farm area decreased between 1988 (779.57 ha), 1998 (446.59 ha) and 2008 (411.96 ha), but almost bounced back to original levels in 2018 (756.58 ha).

LULC transition matrices can be used to understand the specific changes in Pyin Oo Lwin (Table 3). Transition matrices clearly show the large-scale conversion of green spaces into built-up land. Approximately, 558.90 ha of urban forests, and 489.17 ha of seasonal farms were converted to built-up area between 1988 and 1998. The conversion of urban forests and seasonal farms to built-up land accelerated in the next two decades, with 657.76 ha and 297.43 ha of urban forests and seasonal farms lost respectively between 1998 and 2008, and 1246.65 ha and 518.20 ha lost respectively between 2008 and 2018. In total, 1536.07 ha of urban forests and 1229.07 ha of seasonal farms were converted into built-up land within three decades.

**Table 3:** Land use change transition matrices (ha).

	(a) 1988–1998								
	**1998**								
		Water	Built-up land	Urban forest	Urban agriculture (seasonal farms)	Urban agriculture (coffee farms)	Grass land	Other	**Total**
**1988**	Water	19.41	0.00	3.07	0.00	1.58	0.00	5.53	29.58
	Built-up land	9.76	340.95	250.02	51.70	18.33	8.53	51.36	730.64
	Urban forest	5.13	**558.90**	3761.23	**485.93**	**192.48**	192.52	141.52	5337.71
	Urban agriculture (seasonal farms)	2.76	**489.17**	**1026.13**	1004.70	**102.43**	177.82	163.52	2966.53
	Urban agriculture (coffee farms)	0.00	50.45	373.51	165.10	129.06	7.97	53.48	779.57
	Grass land	0.00	24.21	36.23	84.00	1.71	19.59	15.04	180.78
	Other	0.00	35.50	16.39	54.48	1.00	19.73	7.92	135.02
	**Total**	**37.04**	**1499.18**	**5466.58**	**1845.91**	**446.59**	**426.16**	**438.37**	**10159.82**
	(b) 1998–2008								
	**2008**								
		Water	Built-up land	Urban forest	Urban agriculture (seasonal farms)	Urban agriculture (coffee farms)	Grass land	Other	**Total**
**1998**	Water	30.54	1.47	4.08	0.00	0.24	0.00	0.70	37.04
	Built-up land	0.00	853.28	401.09	136.66	7.32	38.71	62.11	1499.18
	Urban forest	0.69	**657.76**	3395.94	**886.08**	**279.51**	144.36	102.24	5466.58
	Urban agriculture (seasonal farms)	0.00	**297.43**	525.99	772.74	**44.09**	110.85	94.81	1845.91
	Urban agriculture (coffee farms)	0.00	46.73	238.58	68.54	62.15	2.75	27.84	446.59
	Grass land	0.00	53.03	186.45	118.11	1.67	49.32	17.58	426.16
	Other	5.98	146.15	149.51	75.81	16.98	6.27	37.67	438.37
	**Total**	**37.21**	**2055.85**	**4901.63**	**2057.94**	**411.96**	**352.27**	**342.96**	**10159.82**
	(c) 2008–2018								
	**2018**								
		Water	Built-up land	Urban forest	Urban agriculture (seasonal farms)	Urban agriculture (coffee farms)	Grass land	Other	**Total**
**2008**	Water	33.65	2.85	0.67	0.00	0.04	0.00	0.00	37.21
	Built-up land	2.87	1555.89	238.94	105.31	48.65	26.98	77.21	2055.85
	Urban forest	11.41	**1246.65**	2396.00	**530.95**	**378.27**	174.83	163.51	4901.63
	Urban agriculture (seasonal farms)	0.00	**518.20**	542.97	618.35	**164.52**	116.47	97.43	2057.94
	Urban agriculture (coffee farms)	0.00	33.70	214.86	18.54	138.56	0.83	5.46	411.96
	Grass land	0.00	80.47	113.35	68.44	12.38	52.53	25.10	352.27
	Other	1.00	169.45	51.57	69.12	14.14	12.09	25.59	342.96
	**Total**	**48.94**	**3607.20**	**3558.36**	**1410.71**	**756.58**	**383.73**	**394.30**	**10159.82**
	(d) 1988–2018								
	**2018**								
		Water	Built-up land	Urban forest	Urban agriculture (seasonal farms)	Urban agriculture (coffee farms)	Grass land	Other	**Total**
**1988**	Water	21.23	3.83	2.87	0.00	1.65	0.00	0.00	29.58
	Built-up land	8.03	485.42	162.92	30.76	22.94	6.03	14.54	730.64
	Urban forest	13.06	**1536.07**	2242.97	**658.28**	**507.80**	207.23	172.31	5337.71
	Urban agriculture (seasonal farms)	5.62	**1229.07**	821.85	481.86	**149.70**	132.94	145.48	2966.53
	Urban agriculture (coffee farms)	0.00	256.02	237.57	181.17	61.88	10.85	32.08	779.57
	Grass land	1.00	38.17	59.75	44.95	9.71	13.47	13.73	180.78
	Other	0.00	58.63	30.42	13.69	2.91	13.22	16.15	135.02
	**Total**	**48.94**	**3607.20**	**3558.36**	**1410.71**	**756.58**	**383.73**	**394.30**	**10159.82**

Figures in bold indicate the most important results, some of which are discussed in the main text.

Interestingly, apart from the extensive loss of LULC classes associated with green spaces to built-up land, there were also instances of transformation between such LULC classes (Table 3). For example, large portions of urban forest were transformed into urban farms and coffee plantations. Approximately, 485.93 ha, 886.08 ha, and 530.95 ha of urban forests were converted to seasonal farms between 1988–1998, 1998–2008, and 2008–2018 respectively. Additionally, 192.48 ha, 279.51 ha, and 378.27 ha of urban forests were changed into coffee farms between 1988–1998, 1998–2008, and 2008–2018 respectively. Interestingly there is a large conversion of urban farms to urban forest. For example, approximately 1026.13 ha of urban farms were transformed into urban forest between 1988 and 1998. One of the possible reasons is the promotion of community forestry (CF) practices in Myanmar that starting in the early-mid 1990s. According to personal communication with the local forest department, Pyin Oo Lwin was one of the target regions, with farmers provided with incentives by the government to establish CF for the production and commercialization of forest and farm products.

### Tree species diversity

We identified a total of 82 different tree species from 35 families in the different urban green spaces. The botanical gardens contain 32 species (from 20 families), the monasteries contained 73 species (from 35 families), the coffee farms contained 12 species (from 10 families), and the city golf course contained 10 species (from 10 families).

The most dominant large tree species (DBH≥5 cm, H>1.3 m) were *Lithocarpus dealbatus* (IVI = 66.22), *Lithocarpus dealbatus* (IVI = 29.84), *Grevillea robusta* (IVI = 192.71), and *Acacia auriculiformis* (IVI = 17.88) in the botanical garden, monasteries, coffee farms, and the city golf course, respectively. The most dominant small tree species (DBH<5 cm, H>1.3 m) were *Litsea glutinosa* (IVI = 48.94), *Bambusa tulda* (IVI = 23.95), and *Coffea arabica* (IVI = 254.42) in the botanical gardens, monasteries, and coffee farms, respectively (there was an absence of small trees in the golf course). The top ten dominant species with the highest IVI scores are summarized in S5 Table.

In terms of the Shannon diversity index, the diversity for large trees (DBH≥5 cm, H> 1.3 m) ranged substantially between green spaces: botanical garden (2.59), monasteries (3.43), coffee farms (1.14), and the golf course (1.57). Similarly, large ranges were also observed and for the diversity of small trees (DBH<5 cm, H>1.3 m): botanical garden (2.58), monasteries (3.17), coffee farms (0.26), and golf course (0). Scores for each plot are included in S6 Table.

### Carbon stocks

**Fig 3:**
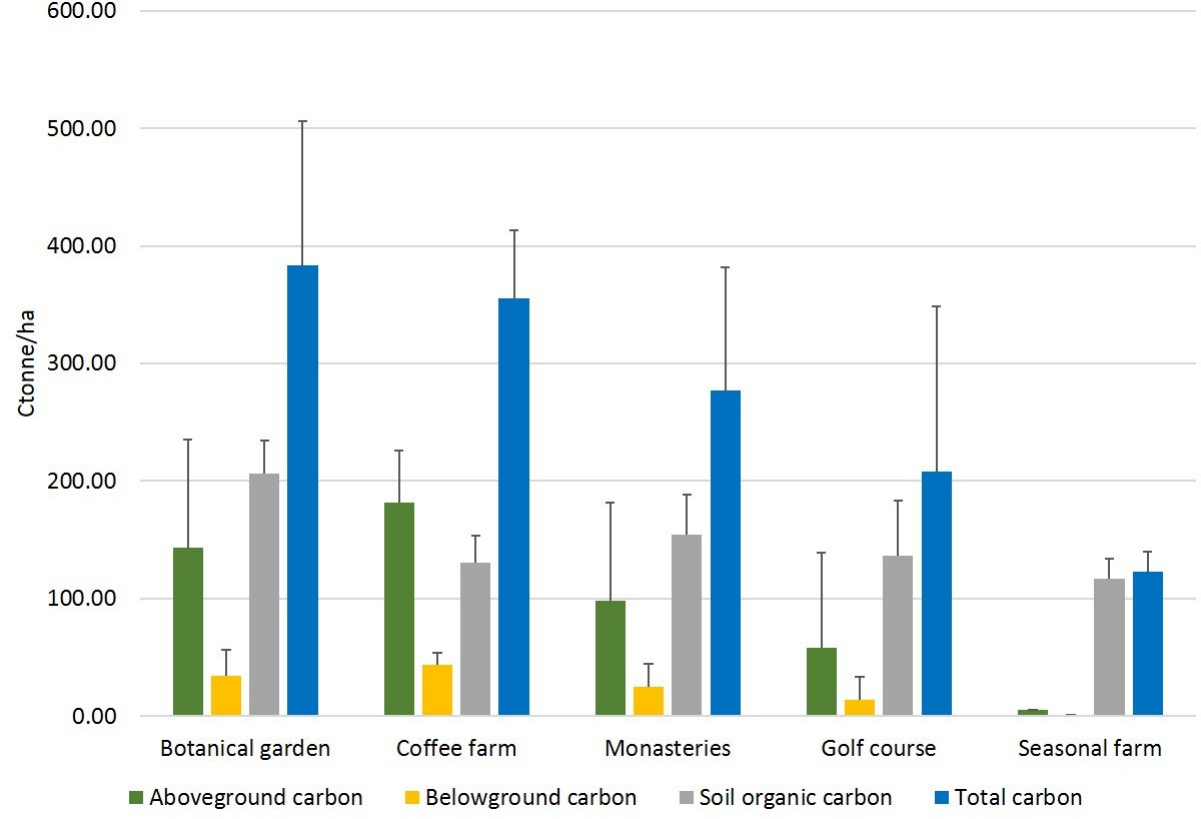
Carbon stocks in different urban green spaces.

Overall the highest aboveground carbon stocks were estimated in coffee farms (181.98±83.35 t/ha), followed by the botanical garden (142.90±92.76 t/ha), monasteries (97.90±43.69 t/ha), and the golf course (58.23±80.84 t/ha) (Table 4, Fig 3). As already mentioned, the aboveground biomass of seasonal farms was assumed at 5 t/ha (see section: Carbon stock estimation). The largest below ground carbon stocks also occurred in the coffee farms (43.53±20.04 t/ha) followed by the botanical gardens (34.17±22.02 t/ha), monasteries (24.60±10.73 t/ha), golf course (13.71±19.40 t/ha) (Table 4, Fig 3). Based on the above assumption the belowground carbon content of seasonal farms was estimated at 1.2 t/ha. In terms of SOC, the botanical garden had the largest stocks (206.61±28.03 t/ha), followed by the monasteries (154.65±23.68 t/ha), golf course (136.50±46.94 t/ha), coffee farms (130.13±34.24 t/ha) and the seasonal farms (117.02±16.59 t/ha) (Table 4, Fig 3). Data for each plot are included in S6 Table.

**Table 4:** Carbon storage in different urban green spaces.

Green space category	Above ground biomass (t/ha)	Below ground biomass (t/ha)	Soil organic carbon (t/ha)	Total carbon (t/ha)
Botanical garden	142.90 ± 92.76	34.17 ± 22.20	206.61 ± 28.03	**383.67 ± 122.47**
Coffee farms	181.98 ± 83.35	43.53 ± 20.04	130.13 ± 34.24	**355.64 ± 104.63**
Monasteries	97.90 ± 43.69	24.60 ± 10.73	154.65 ± 23.68	**277.14 ± 57.37**
Golf course	58.23 ± 80.84	13.71 ± 19.40	136.50 ± 46.94	**208.45 ± 140.59**
Seasonal farms	5.00	1.20	117.02 ± 16.59	**123.22 ± 16.59**

The above ground carbon storage of seasonal farms was assumed based on [84].

Overall the botanical garden was the largest carbon reservoir (383.67 t/ha), followed by coffee farms (355.64 t/ha), the monasteries (277.14 t/ha), golf course (208.45 t/ha), and seasonal farms with (123.22 t/ha) (S7 Table).

**Fig 4:**
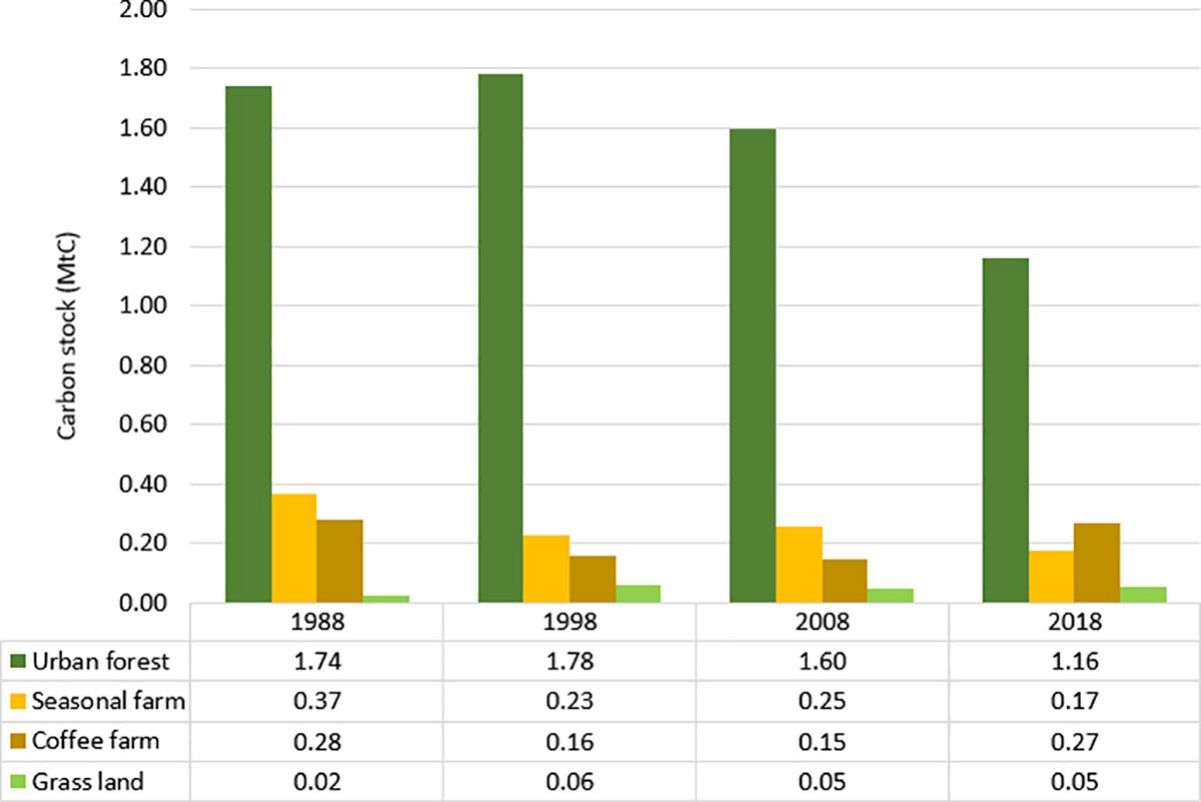
Total carbon stocks change between 1988 and 2018.

To check whether there is a significant difference in carbon stocks between the different LULC classes we use the Kruskal-Wallis test for non-parametric distribution. The results suggest the existence of significant differences in carbon storage between the different LULC classes (S8 and S9 Tables). Subsequently we conducted multiple pairwise comparisons between the carbon stocks of the different LULC classes using Dunn’s test with Bonferroni adjustment to establish whether these differences are statistically significant (Table 5). The overall results suggest that total carbon stocks of urban forest and coffee farm plots are significantly higher (p<0.01) compared to those of plots in seasonal farms and grassland. In the remaining urban green spaces, approximately 1.65 Mt of carbon is stored (Fig 4).

**Table 5:** Differences between mean carbon stocks of different land use and land cover classes.

	Urban forests	Coffee farms	Seasonal farms
**Aboveground carbon stock**			
Coffee farms	-1.95 (1.000)		
Seasonal farms	5.63*** (0.000)	4.75*** (0.000)	
Grassland	4.85*** (0.000)	4.79*** (0.000)	1.64 (0.303)
**Belowground carbon stock**			
Coffee farms	-0.90 (1.000)		
Seasonal farms	5.65*** (0.000)	4.72*** (0.000)	
Grassland	4.86*** (0.000)	4.77*** (0.000)	1.64 (0.303)
**Soil organic carbon stock**			
Coffee farms	2.22 (0.079)		
Seasonal farms	4.07*** (0.000)	0.56 (1.000)	
Grassland	1.79 (0.220)	-0.04 (1.000)	-0.51 (1.000)
**Total carbon stock**			
Coffee farms	-0.52 (1.000)		
Seasonal farms	5.74*** (0.000)	4.40*** (0.000)	
Grassland	3.03*** (0.007)	2.94*** (0.009)	-0.22 (1.000)

Significant level indicates

***p<0.01

**p<0.05

*p<0.1.

## Discussion

The LULC change analysis (see section: Land use and land cover change) clearly shows that the built-up area of Pyin Oo Lwin administrative area expanded significantly in the past three decades following the 1988 market economic liberalization policy. The expansion of built up land has largely been at the expense of urban and peri-urban forest and agricultural land, both within the city and its periphery. These rates accelerated even more the decade after the implementation of the economic liberalization policies with the city expanding in all directions between 1998–2008. Substantial, but less pronounced, expansion has also been observed since 2008 following the constitution law that made the development of urban areas a responsibility of the individual states and regions of Myanmar, rather than the national government. Within three decades, green spaces shrunk considerably, with built-up areas now occupying a large portion of the administrative area (35.50%).

Most of the built-up land expansion can be attributed to rural-urban migration commonly observed in other Myanmar cities (see section: Introduction). However, the expansion of built-up land in Pyin Oo Lwin has also been partly fuelled by tourism, especially in the past decade. Due to its rich colonial history and accessibility to scenic landscapes, the city is one of the most famous tourist destinations in Myanmar. For example, 1,839,823 people visited the botanical gardens in 2017 [89]. Expert interviews with local authorities highlighted that many wealthy citizens from other parts of Myanmar have been investing in real estate (usually second or third homes) to capitalize on the city’s favorable climate and abundant recreational options (pers. comm., Sein Lann Pyin Oo Lwin).

**Fig 5:**
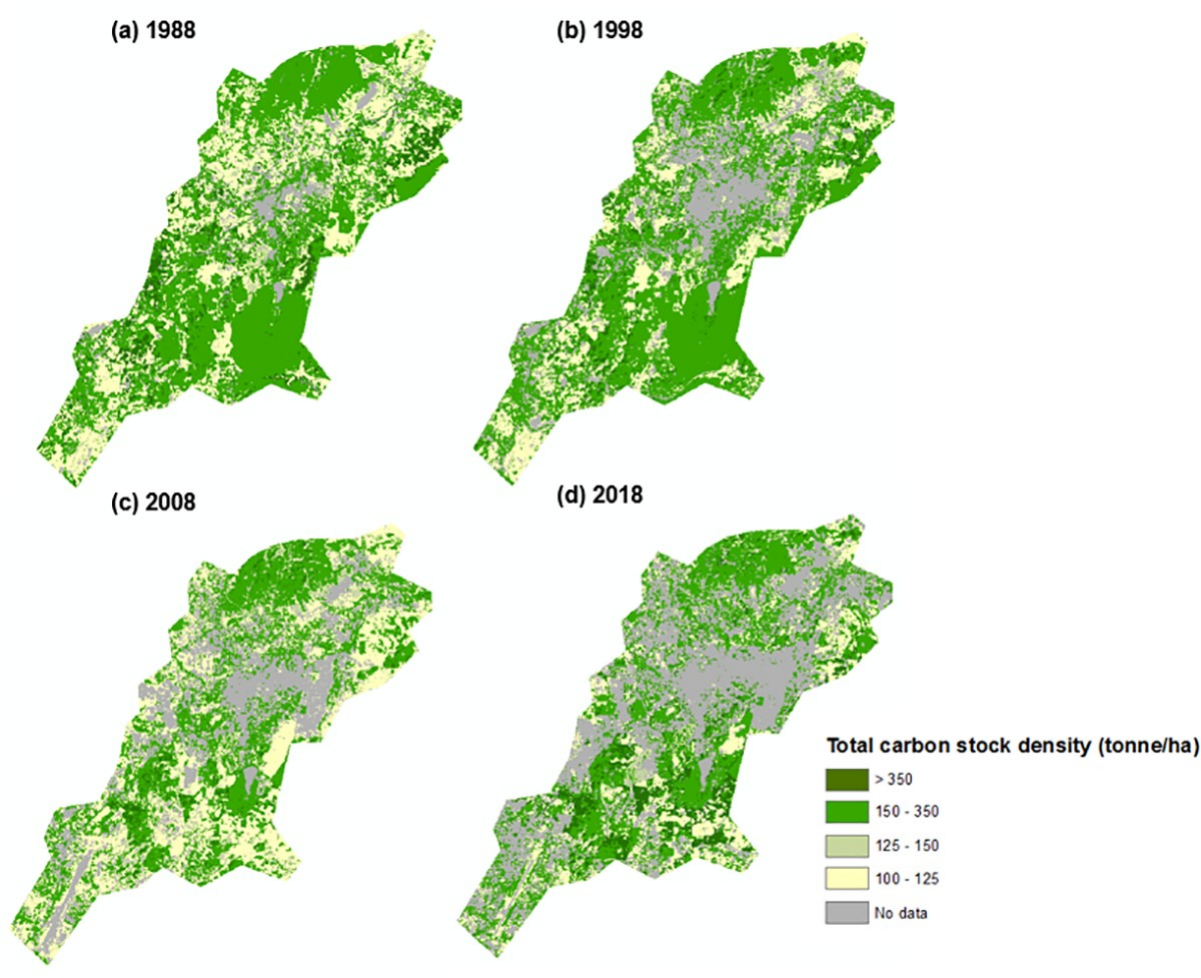
Total carbon stock density in Pyin Oo Lwin (t/ha).

However, regardless of the underlying demographic and socioeconomic factors, the loss of the green spaces can have important ramifications for the current policy efforts to foster green economic transitions in Myanmar [23,90]. Indeed most green spaces in the city can store substantial amounts of carbon (Fig 5) and be important habitats for tree species.

In particular, all green spaces contain significant carbon stocks. The botanical garden contains the highest total carbon stocks (383.67 t/ha), followed by the coffee farms (355.64 t/ha), monasteries (277.14 t/ha), golf course (208.45 t/ha) and seasonal farms (123.22 t/ha). This suggests that green spaces dominated by forest remnants (i.e. botanical gardens, monasteries), trees (i.e. coffee farms) and well-maintained grassland (i.e. golf course) exhibit high carbon storage potential. This reflects many other studies in developing and developed countries that have quantified the carbon storage and/or sequestration services derived from urban forests [31,91,92] and grassland in golf courses [93]. However, the extensive LULC change associate with uncontrolled urbanization in the past three decades reduced substantially the carbon stocks within the city administrative area, from 2.41 Mt (1988) to 1.65 Mt (2018) (Fig 4). We have to point, however, that these results should be treated with caution as past carbon storage estimates are uncertain as we assume tree species composition that is the same as in 2018 (see section: Carbon stock estimation).

Similarly, some green spaces, and especially those dominated by urban forests contain many tree species that characterize the main forest type in the area (Hill Evergreen Forest). We identified 82 different tree species from 35 families across the different urban green spaces, with tree diversity being particularly high in monasteries. This suggests that such green spaces can be particularly valuable for maintaining native biodiversity in this rapidly urbanising context. It is also interesting to note that tree species diversity has a weak positive but statistically significant correlation with carbon stocks (r = 0.56, p<0.01) (see background data in S6 Table). Thus preserving the existing and developing new green spaces that contain diverse tree species could in theory provide substantial carbon storage benefits, in addition to many other ecosystem services (see section: Introduction) [94]. This reflects other studies and meta-analyses that have found positive associations between biodiversity and carbon storage and/or sequestration [94,95].

Green infrastructure consisting of green spaces such as the ones studied in this paper could provide multiple benefits to urban residents, contributing to current national policy goals related to urban sustainability and transitioning to a green economy (see section: Introduction). However, understanding better the green economic potential of green space maintenance/development and green infrastructure in Pyin Oo Lwin (and more broadly in Myanmar) and realizing it, would require substantial efforts from scientists, local government, and other stakeholders.

First, the research community should provide better estimates of the actual climate change mitigation potential of green spaces, both at the scale of the individual green space and the city as a whole. This would require accurate assessments of carbon sequestration capacity and GHG emissions associated with management options such as fertilizer/pesticides use, irrigation, and energy use for mowing and other landscaping activities, among others. There is an emerging literature on these topics [96–100], but practically no relevant studies in developing countries such as Myanmar. Subsequently, the net-storage capacity of urban green spaces at the city scale should be compared with the total urban GHG emissions to assess the actual mitigation potential of green spaces at the city level, and thus their ability to contribute to green economic transitions. Again, this would require substantial research effort that is compounded largely by the current lack of (and low capacity to provide) accurate GHG emission inventories at the city scale in Myanmar (pers. Comm. Department of Environmental Conservation).

Second, at the local government level it would be important to build capacity between different departments to design green spaces and develop and implement appropriate urban plans. The latter refers to urban plans for both creating green infrastructure and reconciling land demand between different competing urban activities. This capacity is currently lacking in most cities of Myanmar and especially the smaller secondary cities. It would require, among others, the training of city officials and a closer interaction with national and international research institutions. This lack of capacity was attested by many practitioners both at the city and the national level (pers. Comm. Sein Lann Pyin Oo Lwin).

Finally, when it comes to collaboration between city governments and other stakeholders it is important to note that most urban forest remnants are preserved within Buddhist monasteries and military compounds. In the former case, the active participation of religious institutions would be important for ensuring the proper management of urban forest areas in existing monasteries. In the latter case, a strong coordination with military agencies would be necessary to ensure that following the possible decommission of these camps, substantial portions of the urban forests will be used for the development of green spaces.

## Conclusions

This study focused on the LULC change effects of urbanization in Pyin Oo Lwin, a secondary city of Myanmar. LULC change analysis based on remote sensing shows the significant expansion of urban built-up land at the expense of urban green spaces such as urban/peri-urban forests and agricultural land. Specifically, by 2018, 35.50% of the city’s administrative area consisted of built-up area (up from 7.19% in 1988), with the remaining covered mainly by a combination of urban forests (35.02%), urban agricultural areas (21.33%) and grassland (3.78%).

The different green spaces scattered around the city store substantial amounts of carbon, especially in urban forests (i.e. urban park, monasteries), agroforestry systems (i.e. coffee farms), and well-maintained grassland (i.e. golf course). Areas with higher tree species diversity tend to contain higher carbon stocks, suggesting a positive relationship between tree species diversity and carbon storage. However, the uncontrolled urbanization has resulted in the loss of urban green spaces, and as an extent of carbon stocks between 1988 (2.41 Mt) and 2018 (1.65 Mt).

Our study makes the case that urban green spaces can provide important regulating services related to carbon storage. Such ecosystem services can contribute manifold to ongoing efforts to foster sustainable urban development and green economic transitions in the country. However, there is low capacity in many of Myanmar’s smaller cities to achieve this. In this respect it is urgently required to develop urban policies and plans. Developing an evidence base, raising awareness, preserving existing urban green spaces, and developing green infrastructure are all important aspects for the future urbanization transition in the country.

## Supporting information

S1 Fig Location of aboveground biomass surveys and soil sampling sites in each green space. (TIF)

S2 Fig Vegetation and soil sampling plot design for urban forest area. (TIF)

S3 Fig Soil sample plot sampling strategy for different urban farm sizes: (a) 1000 m^2^, (b) <100 m^2^, (c)(d) are 100–1000 m^2^.
(TIF)

S1 Table Landsat images used for land use and land cover (LULC) change analysis. (DOCX)

S2 Table Land use type description. (DOCX)

S3 Table Allometric equations for carbon estimation in vegetation. (DOCX)

S4 Table Error matrix for land use classification (1988, 1998, 2008, and 2018). (DOCX)

S5 Table Tree species diversity in different urban green spaces. (DOCX)

S6 Table Carbon stocks component and Shannon diversity index for each plot. (DOCX)

S7 Table Carbon stock for each green space. (DOCX)

S8 Table Summary statistics for carbon stock components. (DOCX)

S9 Table Kruskal-Wallis test for carbon stock components. (DOCX)
