# Supplementary figures and images for: Land use change, carbon stocks and tree species diversity in green spaces of a secondary city in Myanmar, Pyin Oo Lwin
Source: PLoS One. 2019 Nov 26;14(11):e0225331. doi: 10.1371/journal.pone.0225331 (PMC6879162; doi:10.1371/journal.pone.0225331)

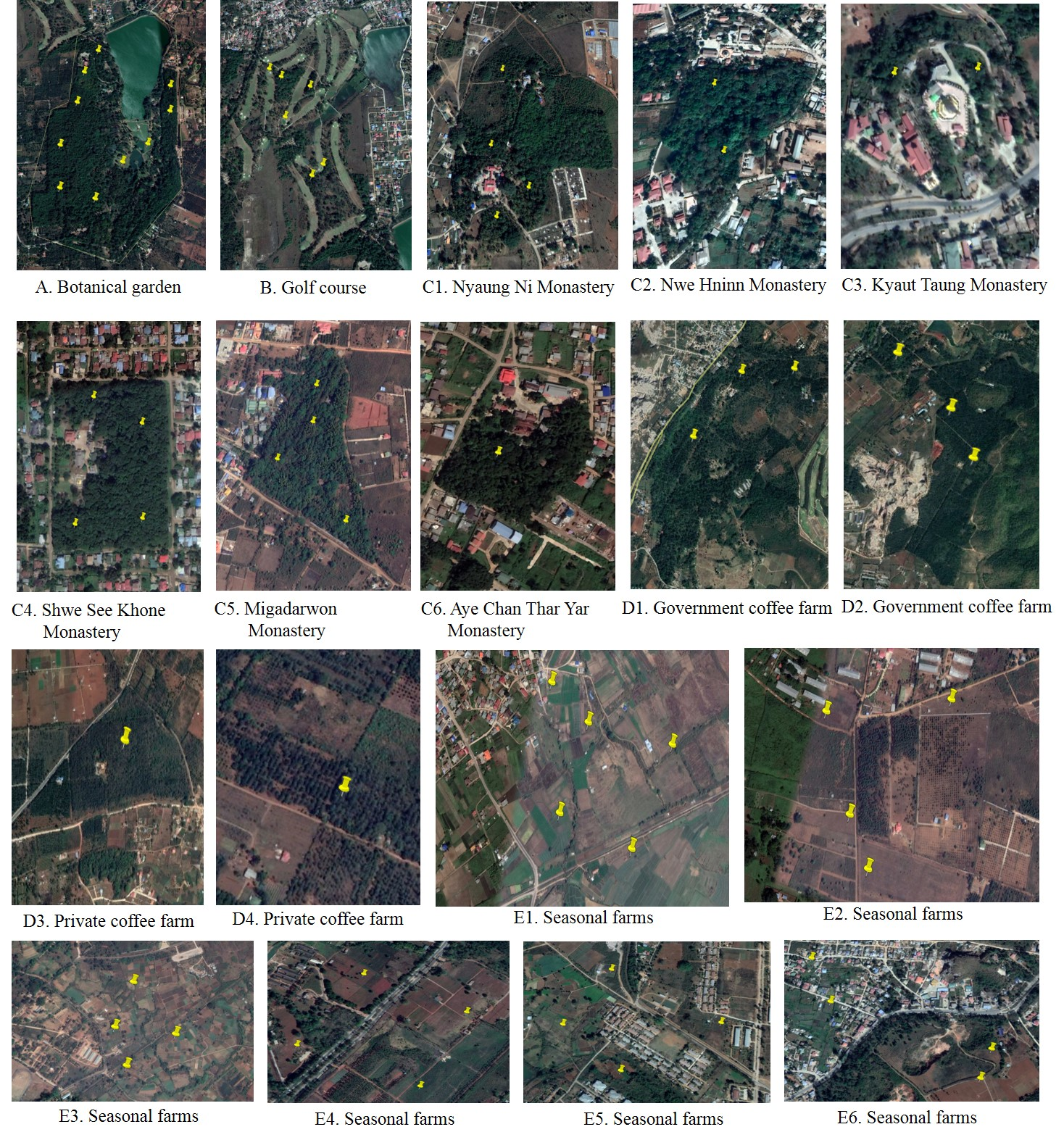

Supplement: S1 Fig — (TIF) [file pone.0225331.s001.tif]

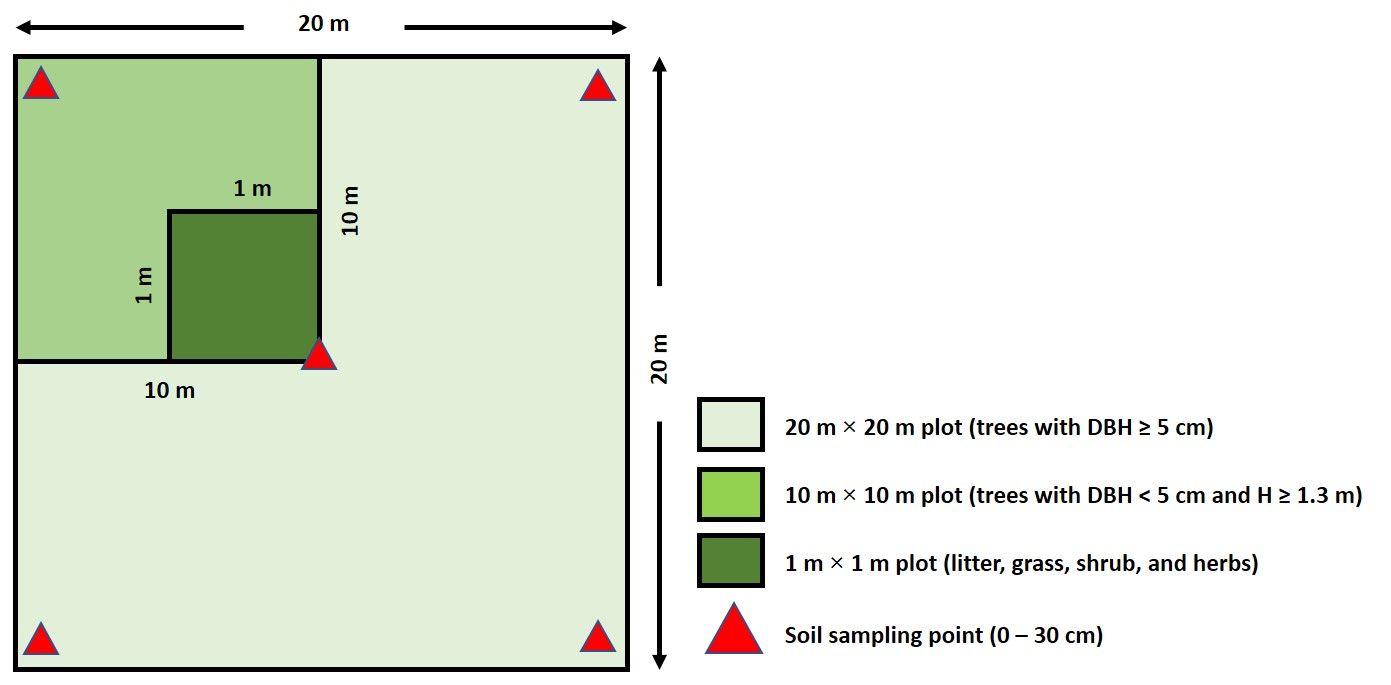

Supplement: S2 Fig — (TIF) [file pone.0225331.s002.tif]

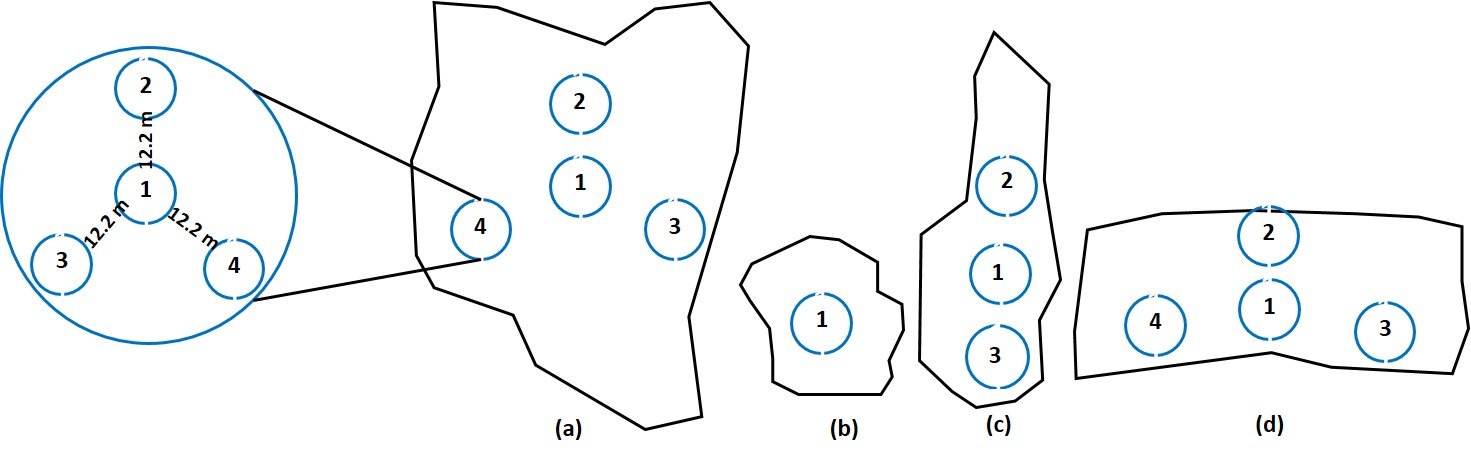

Supplement: S3 Fig — Soil sample plot sampling strategy for different urban farm sizes: (a) 1000 m2, (b) <100 m2, (c)(d) are 100–1000 m2. (TIF) [file pone.0225331.s003.tif]
