# Supplementary material for: Land use change, carbon stocks and tree species diversity in green spaces of a secondary city in Myanmar, Pyin Oo Lwin
Source: PLoS One. 2019 Nov 26;14(11):e0225331. doi: 10.1371/journal.pone.0225331 (PMC6879162; doi:10.1371/journal.pone.0225331)
Supplement: S1 Table — (DOCX) [file pone.0225331.s004.docx]

S1 Table. Landsat images used for land use and land cover (LULC) change analysis

| Data | Sensor Identifier | Data type | Resolution (m) | Acquisition date |
| --- | --- | --- | --- | --- |
| Landsat 8 | Operational land imager-OLI | OLI_TIRS_L1TP | 30 | 25 Feb 2018 |
| Landsat 5 | Thematic Mapper-TM | TM_L1TP | 30 | 14 Feb 2008 |
| Landsat 5 | Thematic Mapper-TM | TM_L1TP | 30 | 02 Feb 1998 |
| Landsat 5 | Thematic Mapper-TM | TM_L1TP | 30 | 23 Feb 1988 |
