# Supplementary material for: Land use change, carbon stocks and tree species diversity in green spaces of a secondary city in Myanmar, Pyin Oo Lwin
Source: PLoS One. 2019 Nov 26;14(11):e0225331. doi: 10.1371/journal.pone.0225331 (PMC6879162; doi:10.1371/journal.pone.0225331)
Supplement: S2 Table — (DOCX) [file pone.0225331.s005.docx]

S2 Table. Land use type description

| **Land use and cover type** | **NDVI value** | **Description** |
| --- | --- | --- |
| Water | -0.14 to -0.26 | Area covered by with water such as lake, pond and dam |
| Built-up land | 0.22 to 0.23 | Area with residential, commercial, transportation and facility developments |
| Urban forest | 0.78 to 0.79 | Area covered predominately with trees. These areas usually contain fragments of (often degraded) forest encroached by built-up land and agricultural activities. |
| Urban agriculture (seasonal farms) | 0.26 to 0.32 | Mainly family farms that grow predominately seasonal crops, vegetables, and flowers, for subsistence purposes or selling to markets. |
| Urban agriculture (coffee farms) | 0.61 to 0.71 | Coffee plantation, either private or operated by government |
| Grass land | 0.53 to 0.60 | Area covered with grass (usually associated with lawns, grazing areas, and possibly abandoned agricultural land) |
| Other | 0.06 to 0.14 | Other land use and cover |
