# Supplementary material for: Land use change, carbon stocks and tree species diversity in green spaces of a secondary city in Myanmar, Pyin Oo Lwin
Source: PLoS One. 2019 Nov 26;14(11):e0225331. doi: 10.1371/journal.pone.0225331 (PMC6879162; doi:10.1371/journal.pone.0225331)
Supplement: S3 Table — (DOCX) [file pone.0225331.s006.docx]

S3 Table. Allometric equations for carbon estimation in vegetation

| **No** | **Family name** | **Species name** | **Local name** | **Biomass equation** | **Selection status** | **Component** | **Reference** |
| --- | --- | --- | --- | --- | --- | --- | --- |
| 1 | Anacardiaceae | *Mangifera indica* L. | Thayet (Mango) | Y=(0.148*((H)^(2)))^(2.407) | similar family | B Bd Bg Bt L S T | (Appuhamy, Samarappuli & Karunaratne, 2009) |
| 2 | Anacardiaceae | *Melanorrhoea usitata* Wall. | Thit sae | Y=(0.148*((H)^(2)))^(2.407) | similar family | B Bd Bg Bt L S T | (Appuhamy, Samarappuli & Karunaratne, 2009) |
| 3 | Anacardiaceae | *Spondias mangifera* Willd. | Gwe | Y=(0.148*((H)^(2)))^(2.407) | similar family | B Bd Bg Bt L S T | (Appuhamy, Samarappuli & Karunaratne, 2009) |
| 4 | Anacardiaceae | *Swintonia floribunda* Griff. | Taung-thayet | Y=(0.148*((H)^(2)))^(2.407) | similar genus | B Bd Bg Bt L S T | (Appuhamy, Samarappuli & Karunaratne, 2009) |
| 5 | Annonaceae | *Cananga odorata* (Lam.) Hook. f. & Thomson | Saga-sein | Y=0.0421*((DBH)^(2)*(H))^(0.9440) | similar family | B Bd Bg Bt L S T | (Hung, Son, & Hung, 2012) |
| 6 | Apocynaceae | *Holarrhena pubescens* Wall. ex G. Don | Lettok-gyi | Y=2.846+0.148*(DBH)*(H) | similar family | B Bd Bg Bt L S T | (Toky, Riddell-Black, Harris, Vasudevan, & Davies, 2011) |
| 7 | Apocynaceae | *Rauvolfia microcarpa* Hook. f. | Taung-zalat | Y=2.846+0.148*(DBH)*(H) | similar family | B Bd Bg Bt L S T | (Toky et al., 2011) |
| 8 | Apocynaceae | *Wrightia tomentosa* Roem. & Schult. | Dar tha khing | Y=2.846+0.148*(DBH)*(H) | similar family | B Bd Bg Bt L S T | (Toky et al., 2011) |
| 9 | Bignoniaceae | *Heterophragma adenophylla* (Wall.) Seem. ex Benth. & Hook. | Phet than | Y=0.1277*(DBH)^2.3943 | similar family | B Bd Bg Bt L S T | (Hung et al., 2012) |
| 10 | Bignoniaceae | *Jacaranda acutifolia* Humb. & Bonpl. | Seinban-apya | Y=0.1277*(DBH)^2.3944 | similar family | B Bd Bg Bt L S T | (Hung et al., 2012) |
| 11 | Bignoniaceae | *Oroxylum indicum* (L.) Kurz | Kyaung-sha | Y=0.1277*(DBH)^2.3944 | similar family | B Bd Bg Bt L S T | (Hung et al., 2012) |
| 12 | Bombacaceae | *Bombax ceiba* L. | Letpan | Y= (0.0841*(DBH)^2.41) | similar family | ABG | (Na’var, 2009) |
| 13 | Caesalpiniaceae | *Cassia mimosoides* L. | Mezali | Y=(0.37*(DBH)^1.96) | similar family | ABG | (Návar, 2009b) |
| 14 | Caesalpiniaceae | *Cassia spectabilis* DC. | Panama-ngu | Y=(0.37*(DBH)^1.96) | similar family | ABG | (Návar, 2009b) |
| 15 | Calophyllaceae | *Mesua ferrea* L. | Gangaw | Y=0.222*(DBH)^(2.387) | similar genus | B Bd Bg Bt L S T | (Puong et al., 2012) |
| 16 | Combretaceae | *Combretum acuminatum* Roxb. | Nabu-nwe | Y=0.0670*((DBH)^(2.5915)) | similar family | B Bd Bg Bt L S T | (Hung, N.D., Giang, L.T., Tu, D.N., Hung, P.T., Lam, P.T., Khanh, N.T., Thuy, 2012) |
| 17 | Combretaceae | *Terminalia bellerica* Roxb. | Thit-seint | Y=0.0670*((DBH)^(2.5915)) | similar genus | B Bd Bg Bt L S T | (Hung, N.D., Giang, L.T., Tu, D.N., Hung, P.T., Lam, P.T., Khanh, N.T., Thuy, 2012) |
| 18 | Combretaceae | *Terminalia chebula* Retz. | Pan ga | Y=0.0670*((DBH)^(2.5915)) | similar genus | B Bd Bg Bt L S T | (Hung, N.D., Giang, L.T., Tu, D.N., Hung, P.T., Lam, P.T., Khanh, N.T., Thuy, 2012) |
| 19 | Cupressaceae | *Cupressus goveniana* var. knightiana Rehd. | Pyin-oo-hwin | Y=1.089*((DBH)^1.6481) | similar genus | B Bd Bg Bt L S T | (Sharma, 2011) |
| 20 | Datiscaceae | *Tetrameles nudiflora* R. Br. | Thit-pok | Y=42.69-12.800*DBH+1.424*DBH^2 | same sp. | ABG | (Chheng et al., 2016) |
| 21 | Dilleniaceae | *Dillenia aurea* Sm. | Metkauk | Y=111.293+0.00004*(DBH)^(2)*(H) | similar genus | B Bd Bg Bt L S T | (Rai, 1984) |
| 22 | Ericaceae | *Craibiodendron stellatum* W.W. Sm. | U-byat | Y=0.018*(DBH)^(3.199) | similar family | B Bd Bg Bt L T | (Perez L.M., Timote, 2010) |
| 23 | Euphorbiaceae | *Croton oblongifolius* Roxb. | Thetyin-gyi | Y=0.1142*(DBH)^(2.4451) | similar family | B Bd Bg Bt L S T | (Hung, N.D., Bay, N.V., Binh, N.D., Tung, 2012) |
| 24 | Euphorbiaceae | *Glochidion velutinum* Wight | Buzo | Y=0.1142*(DBH)^(2.4451) | similar family | B Bd Bg Bt L S T | (Hung, N.D., Bay, N.V., Binh, N.D., Tung, 2012) |
| 25 | Euphorbiaceae | *Mallotus philippinensis* (Lam.) Muell. Arg. | Taw-thi-din | Y=0.1142*(DBH)^(2.4451) | similar genus | B Bd Bg Bt L S T | (Hung, N.D., Bay, N.V., Binh, N.D., Tung, 2012) |
| 26 | Euphorbiaceae | *Sapium baccatum* Roxb. | Ye-badon | Y=exp(-2.24267+2.47464*log((DBH))) | similar genus | B Bd Bg Bt L S T | (Bao Huy et al., 2012) |
| 27 | Fabaceae | *Acacia auriculiformis* A.Cunn.ex Benth. | Acacia | Y=0.078*((((DBH)^2)*(H))^0.902) | same sp. | B Bg Bt L T | (Krisnawati, H., 2012) |
| 28 | Fabaceae | *Albizia odoratissima* (L. f.) Benth. | Taung-magyi | Y=0.0353+0.4055*(DBH)^(2)*(H) | similar genus | Bd Bg Bt L S T | (Chaturvedi & Behl, 1996) |
| 29 | Fabaceae | *Dalbergia cultrata* Grah. | Yin-daik | Y=0.667*(DBH)^(1.832) | similar genus | B Bd Bg Bt L S T | (Ajit, Rai, Singh, & Jabeen, 2010) |
| 30 | Fabaceae | *Erythrina variegata* L. | Kathit | Y=(0.087710)*((DBH)^2.13)*((H)^0.36) | similar family | B Bg Bt L T | (Micosa Tandug, 1987) |
| 31 | Fabaceae | *Leucaena leucocephala* (Lam.) De Wit | Bawzagaing | Y=0.0353+0.4055*(DBH)^(2)*(H) | similar family | Bd Bg Bt L S T | (Chaturvedi & Behl, 1996) |
| 32 | Fabaceae | *Millettia eriocalyx* Dunn | Win-u | Y=0.0353+0.4055*(DBH)^(2)*(H) | similar family | Bd Bg Bt L S T | (Chaturvedi & Behl, 1996) |
| 33 | Fabaceae | *Millettia macrostachya* Collett & Hemsl. | Ye-thinwin | Y=0.0353+0.4055*(DBH)^(2)*(H) | similar family | Bd Bg Bt L S T | (Chaturvedi & Behl, 1996) |
| 34 | Fabaceae | *Millettia pendula* Benth. | Thin-win | Y=0.0353+0.4055*(DBH)^(2)*(H) | similar family | Bd Bg Bt L S T | (Chaturvedi & Behl, 1996) |
| 35 | Fabaceae | *Pterocarpus macrocarpus* Kurz | Thit-padauk | Y=0.0530*(((DBH)^(2))*((H)^(0.7)))^(1.0072) | same sp. | B Bd Bg Bt L S T | (Hung, N.D., Bay, N.V., Binh, N.D., Tung, 2012) |
| 36 | Fagaceae | *Castanopsis indica* A. DC. | Thit-e-gyin | Y=0.1142*(DBH)^(2.4451) | similar genus | B Bd Bg Bt L S T | (Hung, N.D., Bay, N.V., Binh, N.D., Tung, 2012) |
| 37 | Fagaceae | *Lithocarpus dealbatus* (Hook. f. & Thoms. ) | Kywet-sa-ni | Y=0.222*(DBH)^(2.387) | similar genus | B Bd Bg Bt L S T | (Puong et al., 2012) |
| 38 | Fagaceae | *Lithocarpus fenestrata* (Roxb.) Rehd. | Thit-payaung | Y=0.222*(DBH)^(2.387) | similar genus | B Bd Bg Bt L S T | (Puong et al., 2012) |
| 39 | Fagaceae | *Lithocarpus lindleyanus* (Wall.) A. Camus | Phet-kyan | Y=0.222*(DBH)^(2.387) | similar genus | B Bd Bg Bt L S T | (Puong et al., 2012) |
| 40 | Fagaceae | *Quercus griffithii* Hook. f. & Thoms. ex Miq. | Nyanbo | Y=(0.089*(DBH)^2.5226) | similar genus | ABG | (Návar, 2009b) |
| 41 | Fagaceae | *Quercus helferiana* A. DC. | Yingu-akyi | Y=(0.089*(DBH)^2.5226) | similar genus | ABG | (Návar, 2009b) |
| 42 | Fagaceae | *Quercus mespilifolia* Wall. | Thite | Y=(0.089*(DBH)^2.5226) | similar genus | ABG | (Návar, 2009b) |
| 43 | Fagaceae | *Quercus serrata* Thunb. | Thit-e | Y=(0.089*(DBH)^2.5226) | similar genus | ABG | (Návar, 2009b) |
| 44 | Flacourtiaceae | *Flacourtia indica* (Burm. f.) Merr. | Naywe | Y=173.144*((1+exp(2.956-(0.120*(DBH))))^(-1)) | similar family | B Bd Bg Bt L S T | (Das & Chaturvedi, 2005) |
| 45 | Juglandaceae | *Engelhardtia spicata* Blume | Petsut | Y=0.0547*((DBH)^(2.1148))*((H)^(0.6131)) | similar genus | B Bd Bg Bt L S T | (Hung, N.D., Bay, N.V., Binh, N.D., Tung, 2012) |
| 46 | Lamiaceae | *Gmelina arborea* Roxb. | Yemane | Y= 0.06*(((DBH)^2)*(H))^0.88 | same sp. | B Bg Bt L T | (Krisnawati, H., 2012) |
| 47 | Lauraceae | *Cinnamomum camphora* (L.) Nees & Eberm. | Payok | Y=0.076*(DBH)^(2.41467) | similar family | B Bd Bg Bt L T | (Perez L.M., Timote, 2010) |
| 48 | Lauraceae | *Lindera assamica* Kurz | Karawae | Y=0.076*(DBH)^(2.41467) | similar family | B Bd Bg Bt L T | (Perez L.M., Timote, 2010) |
| 49 | Lauraceae | *Litsea glutinosa* (Lour.) C.B.Rob. | Ondon | Y=0.076*(DBH)^(2.41467) | similar family | B Bd Bg Bt L T | (Perez L.M., Timote, 2010) |
| 50 | Lauraceae | *Persea americana* Mill. | Htawbat(Avocado) | Y=0.076*(DBH)^(2.41467) | similar family | B Bd Bg Bt L T | (Perez L.M., Timote, 2010) |
| 51 | Leguminosae | *Archidendron jiringa* (Jack) Nielsen | Danyin | Y=0.0267*((DBH)^2.8912) | same family | B Bg Bt L T | (Krisnawati, H., 2012) |
| 52 | Leguminosae | *Xylia xylocarpa* (Roxb.) Taub. | Pyinkado | Y=0.0154*(((DBH)^(2))*((H)^(0.7)))^1.1682 | same sp. | B Bd Bg Bt L S T | (Hung et al., 2012) |
| 53 | Lythraceae | *Lagerstroemia parviflora* Roxb. | Kyet-tawsa | Y=0.1277*(DBH)^2.3943 | similar genus | B Bd Bg Bt L S T | (Hung et al., 2012) |
| 54 | Malvaceae | *Kydia calycina* Roxb. | Khwe Tayaw (Dwabok) | Y=0.1245*(DBH)^(2.4163) | similar family | B Bd Bg Bt L S T | (Hung et al., 2012) |
| 55 | Meliaceae | *Cedrela serrata* Royle | Taung-tama | Y=(0.130169*(DBH)^2.34924) | similar family | ABG | (Rodríguez R., J. Jiménez, J. Meza, 2008) |
| 56 | Moraceae | *Artocarpus heterophyllus* Lam. | (Peinne)Jackfruit | Y=0.065*((DBH)^2.282) | same sp. | B Bg Bt L T | (Santos Martin, Navarro-Cerrillo, Mulia, & van Noordwijk, 2010) |
| 57 | Moraceae | *Artocarpus lakoocha* Roxb. | Myauk-laung | Y=0.065*((DBH)^2.282) | similar genus | B Bg Bt L T | (Santos Martin et al., 2010) |
| 58 | Moraceae | *Ficus hispida* L. f. | Kadut | Y=0.1245*(DBH)^(2.4163) | similar genus | B Bd Bg Bt L S T | (Hung, N.D., Bay, N.V., Binh, N.D., Tung, 2012) |
| 59 | Moraceae | *Streblus asper* Lour. | Okhne | Y=0.1245*(DBH)^(2.4163) | similar family | B Bd Bg Bt L S T | (Hung, N.D., Bay, N.V., Binh, N.D., Tung, 2012) |
| 60 | Myrtaceae | *Eucalyptus camaldulensis* Dehnh. | Eu-ca-lit | Y= 10.736+0.023*(DBH)^(2)*(H) | same sp. | B Bd Bg Bt L S T | (Toky et al., 2011) |
| 61 | Myrtaceae | *Psidium guajava* L. | Marlakar (guava) | Y= 10.736+0.023*(DBH)^(2)*(H) | similar family | B Bd Bg Bt L S T | (Toky et al., 2011) |
| 62 | Myrtaceae | *Syzygium cumini* (L.) Skeels | Tabyae-phyu | Y= 10.736+0.023*(DBH)^(2)*(H) | similar family | B Bd Bg Bt L S T | (Toky et al., 2011) |
| 63 | Myrtaceae | *Tristaniopsis burmanica* (Griff.) P.G. Wilson & J.T. Waterh. | Dauk-yat | Y= 10.736+0.023*(DBH)^(2)*(H) | similar family | B Bd Bg Bt L S T | (Toky et al., 2011) |
| 64 | Phyllanthaceae | *Emblica officinalis* Gaertn. | Zibyu | Y=1.415040+0.053909*(DBH)^(2)*(H) | same sp. | B Bd Bg Bt L S T F | (Kumar, George, Jamaludheen, & Suresh, 1998) |
| 65 | Pinaceae | *Pinus insularis* Endl. | Htinyu | Y=(0.2018*(DBH)^2.2907) | similar genus | ABG | (Návar, 2009a) |
| 66 | Poaceae | *Bambusa tulda* Roxb. | Thaik-wa | Y=0.182*(DBH)^(2.16) | similar genus | B Bd Bg Bt L S T | (Puong et al., 2012) |
| 67 | Poaceae | *Dendrocalamus longispathus* (Kurz) Kurz | Wah net | Y=0.182*(DBH)^(2.16) | similar family | B Bd Bg Bt L S T | (Puong et al., 2012) |
| 68 | Poaceae | *Thyrsostachys siamensis* (Kurz ex Munro) | Htiyo-wa | Y=0.182*(DBH)^(2.16) | similar family | B Bd Bg Bt L S T | (Puong et al., 2012) |
| 69 | Proteaceae | *Grevillea robusta* A. Cunn. ex R. Br. | Silver oak | Y=4.06*(DBH)^(1.76) | similar family | B Bd Bg Bt L T | (Âs et al., 1999) |
| 70 | Proteaceae | *Macadamia ternifolia* F.Muell. | Magadamea | Y=4.06*(DBH)^(1.76) | similar family | B Bd Bg Bt L T | (Âs et al., 1999) |
| 71 | Rhamnaceae | *Ziziphus incurva* Roxb. | Sugauk | Y=(0.311733*(DBH)^2.04754) | similar family | ABG | (Rodríguez R., J. Jiménez, J. Meza, 2008) |
| 72 | Rhamnaceae | *Ziziphus rugosa* Lam. | Zi-talaing | Y=(0.311733*(DBH)^2.04754) | similar family | ABG | (Rodríguez R., J. Jiménez, J. Meza, 2008) |
| 73 | Rosaceae | *Prunus cerasoides* D. Don | Cherry | Y=(Exp(-2.76)*(DBH)^2.37) | similar genus | ABG | (Acosta Mireles, 2003) |
| 74 | Rosaceae | *Prunus communis* Huds. | Met-mann | Y=(Exp(-2.76)*(DBH)^2.37) | similar genus | ABG | (Acosta Mireles, 2003) |
| 75 | Rubiaceae | *Coffea arabica* L. | Coffee | Y=(Exp(-0.66)*(DBH)^1.37) | same sp. | ABG | (Acosta Mireles, 2003) |
| 76 | Rubiaceae | *Wendlandia tinctoria* DC. | Thitni | Y=0.0547*((DBH)^(2.1148))*((H)^(0.6131)) | similar genus | B Bd Bg Bt L S T | (Hung, N.D., Bay, N.V., Binh, N.D., Tung, 2012) |
| 77 | Rutaceae | *Micromelum hirsutum* Oliver | Pyidawthein | Y=exp(-2.24267+2.47464*log((DBH))) | similar family | B Bd Bg Bt L S T | (Hung, N.D., Bay, N.V., Binh, N.D., Tung, 2012) |
| 78 | Sapindaceae | *Sapindus rarak* DC. | Kala-kimmun | Y=0.0108*((DBH)^(2.708)) | similar genus | B Bd Bg Bt L S T | (Hung, N.D., Bay, N.V., Binh, N.D., Tung, 2012) |
| 79 | Sapindaceae | *Schleichera oleosa* (Lour.) Oken | Gyo | Y=0.0670*((DBH)^(2.5915)) | similar family | B Bd Bg Bt L S T | (Hung, N.D., Bay, N.V., Binh, N.D., Tung, 2012) |
| 80 | Simaroubaceae | *Harrisonia perforata* Merr. | Sugyin | Y=(-1.979)+2.038*(DBH) | similar family | B Bd Bg Bt L S T | (Toky et al., 2011) |
| 81 | Theaceae | *Anneslea fragrans* Wall. | Pan-ma | Y=exp(1.165+0.514*(log((DBH)^(2)*(H)^(1)))) | similar family | Bd Bg Bt S T | (Sundriyal & Sharma, 1996) |
| 82 | Theaceae | *Schima wallichii* (DC.) Korth. | Thityah | Y=exp(1.165+0.514*(log((DBH)^(2)*(H)^(1)))) | similar family | Bd Bg Bt S T | (Sundriyal & Sharma, 1996) |

Note: B (Bark), Bd (Dead branches), Bg (Gross branches), Bt (Thin branches), L (Leaves), S (Stump), T (Trunk under bark)
