# Supplementary material for: Land use change, carbon stocks and tree species diversity in green spaces of a secondary city in Myanmar, Pyin Oo Lwin
Source: PLoS One. 2019 Nov 26;14(11):e0225331. doi: 10.1371/journal.pone.0225331 (PMC6879162; doi:10.1371/journal.pone.0225331)
Supplement: S4 Table — (DOCX) [file pone.0225331.s007.docx]

S4 Table. Error matrix for land use classification (1988, 1998, 2008, and 2018)

|  | 1. 1988 | | | | | | | |  |  |
| --- | --- | --- | --- | --- | --- | --- | --- | --- | --- | --- |
|  | Reference data | | | | | | | | PA | UA |
| Classified data | Water | Built-up area | Urban forest | Urban agriculture  (seasonal farms) | Urban agriculture  (coffee farms) | Grass land | Other | **Total** | % | % |
| Water | 5 | 0 | 0 | 0 | 0 | 0 | 0 | 5 | 100.00 | 100.00 |
| Built-up area | 0 | 10 | 1 | 1 | 0 | 0 | 0 | 12 | 100.00 | 83.33 |
| Urban forest | 0 | 0 | 40 | 2 | 0 | 0 | 0 | 42 | 89.13 | 95.35 |
| Urban agriculture (seasonal farms) | 0 | 0 | 1 | 23 | 1 | 1 | 0 | 26 | 85.19 | 88.46 |
| Urban agriculture (coffee farms) | 0 | 0 | 3 | 0 | 7 | 0 | 0 | 10 | 87.50 | 70.00 |
| Grass land | 0 | 0 | 0 | 1 | 0 | 1 | 0 | 2 | 50.00 | 50.00 |
| Other | 0 | 0 | 0 | 0 | 0 | 0 | 3 | 3 | 100.00 | 100.00 |
| Total | 5 | 10 | 45 | 27 | 8 | 2 | 3 | 100 |  |  |
| Overall accuracy (%) | 89.11 |  |  |  |  |  |  |  |  |  |
| Kappa value | 0.85 |  |  |  |  |  |  |  |  |  |

|  | 1. 1998 | | | | | | | |  |  |
| --- | --- | --- | --- | --- | --- | --- | --- | --- | --- | --- |
|  | Reference data | | | | | | | | PA | UA |
| Classified data | Water | Built-up area | Urban forest | Urban agriculture  (seasonal farms) | Urban agriculture  (coffee farms) | Grass land | Other | **Total** | % | % |
| Water | 9 | 0 | 0 | 0 | 0 | 0 | 0 | 9 | 100.00 | 100.00 |
| Built-up area | 0 | 11 | 1 | 0 | 0 | 0 | 2 | 14 | 91.67 | 78.57 |
| Urban forest | 0 | 0 | 43 | 1 | 0 | 1 | 0 | 45 | 97.78 | 95.65 |
| Urban agriculture (seasonal farms) | 0 | 1 | 0 | 13 | 0 | 0 | 0 | 14 | 81.25 | 92.86 |
| Urban agriculture (coffee farms) | 0 | 0 | 0 | 0 | 7 | 0 | 0 | 7 | 100.00 | 100.00 |
| Grass land | 0 | 0 | 0 | 1 | 0 | 5 | 0 | 6 | 83.33 | 83.33 |
| Other | 0 | 0 | 0 | 1 | 0 | 0 | 4 | 5 | 66.67 | 80.00 |
| Total | 9 | 12 | 44 | 16 | 7 | 6 | 6 | 100 |  |  |
| Overall accuracy (%) | 92.08 |  |  |  |  |  |  |  |  |  |
| Kappa value | 0.89 |  |  |  |  |  |  |  |  |  |

|  | 1. 2008 | | | | | | | |  |  |
| --- | --- | --- | --- | --- | --- | --- | --- | --- | --- | --- |
|  | Reference data | | | | | | | | PA | UA |
| Classified data | Water | Built-up area | Urban forest | Urban agriculture  (seasonal farms) | Urban agriculture  (coffee farms) | Grass land | Other | Total | % | % |
| Water | 6 | 0 | 0 | 0 | 0 | 0 | 0 | 6 | 85.71 | 100.00 |
| Built-up area | 1 | 20 | 0 | 1 | 0 | 0 | 3 | 25 | 90.91 | 80.00 |
| Urban forest | 0 | 0 | 33 | 2 | 2 | 0 | 0 | 37 | 91.67 | 89.19 |
| Urban agriculture (seasonal farms) | 0 | 1 | 2 | 12 | 0 | 0 | 0 | 15 | 76.47 | 81.25 |
| Urban agriculture (coffee farms) | 0 | 0 | 1 | 0 | 7 | 0 | 0 | 8 | 77.78 | 87.50 |
| Grass land | 0 | 0 | 0 | 1 | 0 | 3 | 0 | 4 | 100.00 | 75.00 |
| Other | 0 | 1 | 0 | 0 | 0 | 0 | 4 | 5 | 57.14 | 80.00 |
| Total | 7 | 22 | 36 | 16 | 9 | 3 | 7 | 100 |  |  |
| Overall accuracy (%) | 85.15 |  |  |  |  |  |  |  |  |  |
| Kappa value | 0.81 |  |  |  |  |  |  |  |  |  |

|  | 1. 2018 | | | | | | | |  |  |
| --- | --- | --- | --- | --- | --- | --- | --- | --- | --- | --- |
|  | Reference data | | | | | | | | PA | UA |
| Classified data | Water | Built-up area | Urban forest | Urban agriculture  (seasonal farms) | Urban agriculture  (coffee farms) | Grass land | Other | **Total** | % | % |
| Water | 8 | 0 | 0 | 0 | 0 | 0 | 0 | 8 | 100.00 | 100.00 |
| Built-up area | 0 | 28 | 0 | 0 | 0 | 0 | 0 | 28 | 100.00 | 100.00 |
| Urban forest | 0 | 0 | 25 | 0 | 0 | 1 | 0 | 26 | 92.59 | 96.15 |
| Urban agriculture (seasonal farms) | 0 | 0 | 0 | 9 | 0 | 0 | 0 | 9 | 100.00 | 100.00 |
| Urban agriculture (coffee farms) | 0 | 0 | 2 | 0 | 14 | 0 | 0 | 16 | 100.00 | 87.50 |
| Grass land | 0 | 0 | 0 | 0 | 0 | 9 | 0 | 9 | 90.00 | 100.00 |
| Other | 0 | 0 | 0 | 0 | 0 | 0 | 4 | 4 | 100.00 | 100.00 |
| Total | 8 | 28 | 27 | 9 | 14 | 10 | 4 | 100 |  |  |
| Overall accuracy (%) | 97.03 |  |  |  |  |  |  |  |  |  |
| Kappa value | 0.96 |  |  |  |  |  |  |  |  |  |
