# Supplementary material for: Land use change, carbon stocks and tree species diversity in green spaces of a secondary city in Myanmar, Pyin Oo Lwin
Source: PLoS One. 2019 Nov 26;14(11):e0225331. doi: 10.1371/journal.pone.0225331 (PMC6879162; doi:10.1371/journal.pone.0225331)
Supplement: S5 Table — (DOCX) [file pone.0225331.s008.docx]

S5 Table. Tree species diversity in different urban green spaces

1. Urban park (Large trees with DBH≥5 cm, H>1.3 m) **(Shannon diversity index H′ = 2.59)**

| **No.** | **Local name** | **Family name** | **Species name** | **Relative density** | **Relative frequency** | **Relative dominance** | **IVI** |
| --- | --- | --- | --- | --- | --- | --- | --- |
| 1 | Kywet-sa-ni | Fagaceae | *Lithocarpus dealbatus* (Hook. f. & Thoms.) | 23.44 | 8.97 | 33.81 | 66.22 |
| 2 | Htinyu | Pinaceae | *Pinus insularis* Endl. | 5.47 | 3.85 | 21.96 | 31.27 |
| 3 | Thit-pha-yaung | Fagaceae | *Lithocarpus fenestrata* (Roxb.) Rehd. | 14.84 | 7.69 | 5.52 | 28.06 |
| 4 | Thityah | Theaceae | *Schima wallichii* (DC.) Korth. | 8.07 | 8.97 | 5.62 | 22.67 |
| 5 | Cherry | Rosaceae | *Prunus cerasoides* D. Don | 6.25 | 8.97 | 5.62 | 20.84 |
| 6 | Karawae | Lauraceae | *Lindera assamica* Kurz | 5.21 | 3.85 | 8.39 | 17.45 |
| 7 | Ondon | Lauraceae | *Litsea glutinosa* (Lour.) C.B.Rob. | 7.55 | 7.69 | 1.20 | 16.44 |
| 8 | Thetyin-gyi | Euphorbiaceae | *Croton oblongifolius* Roxb. | 3.65 | 7.69 | 0.98 | 12.32 |
| 9 | Thite | Fagaceae | *Quercus mespilifolia* Wall. | 6.51 | 3.85 | 1.90 | 12.25 |
| 10 | Taung-magyi | Fabaceae | *Albizia odoratissima* (L. f.) Benth. | 3.65 | 6.41 | 1.35 | 11.41 |
|  | Others |  |  | 15.36 | 32.05 | 13.65 | 61.07 |
|  | **Total** |  |  | **100.00** | **100.00** | **100.00** | **300.00** |

(b) Urban park (Small trees with DBH<5 cm, H>1.3 m) **(Shannon diversity index H′ = 2.58)**

| **No.** | **Local name** | **Family name** | **Species name** | **Relative density** | **Relative frequency** | **Relative dominance** | **IVI** |
| --- | --- | --- | --- | --- | --- | --- | --- |
| 1 | Ondon | Lauraceae | *Litsea glutinosa* (Lour.) C.B.Rob. | 18.29 | 14.71 | 15.94 | 48.94 |
| 2 | Kywet-sa-ni | Fagaceae | *Lithocarpus dealbatus* (Hook. f. & Thoms.) | 13.41 | 8.82 | 13.31 | 35.55 |
| 3 | Pyinkado | Leguminosae | *Xylia xylocarpa* (Roxb.) Taub. | 12.20 | 2.94 | 11.25 | 26.39 |
| 4 | Nabu-nwe | Combretaceae | *Combretum acuminatum* Roxb. | 8.54 | 8.82 | 7.77 | 25.13 |
| 5 | Cherry | Rosaceae | *Prunus cerasoides* D. Don | 1.22 | 2.94 | 18.94 | 23.10 |
| 6 | Thityah | Theaceae | *Schima wallichii* (DC.) Korth. | 8.54 | 8.82 | 5.23 | 22.59 |
| 7 | Thit-pha-yaung | Fagaceae | *Lithocarpus fenestrata* (Roxb.) Rehd. | 6.10 | 5.88 | 6.27 | 18.25 |
| 8 | Karawae | Lauraceae | *Lindera assamica* Kurz | 7.32 | 2.94 | 5.51 | 15.77 |
| 9 | Taung-magyi | Fabaceae | *Albizia odoratissima* (L. f.) Benth. | 3.66 | 5.88 | 3.04 | 12.58 |
| 10 | Petsut | Juglandaceae | *Engelhardtia spicata* Blume | 3.66 | 5.88 | 2.73 | 12.27 |
|  | Others |  |  | 17.07 | 32.35 | 10.00 | 59.43 |
|  | **Total** |  |  | **100.00** | **100.00** | **100.00** | **300.00** |

(c) Monasteries (Large trees with DBH≥5 cm, H>1.3 m) **(Shannon diversity index H′ = 3.43)**

| **No.** | **Local name** | **Family name** | **Species name** | **Relative density** | **Relative frequency** | **Relative dominance** | **IVI** |
| --- | --- | --- | --- | --- | --- | --- | --- |
| 1 | Kywet-sa-ni | Fagaceae | *Lithocarpus dealbatus* (Hook. f. & Thoms.) | 16.16 | 3.85 | 9.84 | 29.84 |
| 2 | Thityah | Theaceae | *Schima wallichii* (DC.) Korth. | 4.80 | 6.04 | 13.02 | 23.86 |
| 3 | Thit-e | Fagaceae | *Quercus serrata* Thunb. | 6.11 | 4.40 | 8.88 | 19.39 |
| 4 | Thit-payaung | Fagaceae | *Lithocarpus fenestrata* (Roxb.) Rehd. | 6.84 | 3.30 | 3.52 | 13.66 |
| 5 | Cherry | Rosaceae | *Prunus cerasoides* D. Don | 3.93 | 3.30 | 5.53 | 12.75 |
| 6 | Thit-e-gyin | Fagaceae | *Castanopsis indica* A. DC. | 3.78 | 3.30 | 5.19 | 12.27 |
| 7 | Ondon | Lauraceae | *Litsea glutinosa* (Lour.) C.B.Rob. | 3.20 | 3.85 | 1.95 | 8.99 |
| 8 | Taung-zalat | Apocynaceae | *Rauvolfia microcarpa* Hook. f. | 3.49 | 2.20 | 3.23 | 8.92 |
| 9 | Taung-magyi | Fabaceae | *Albizia odoratissima* (L. f.) Benth. | 2.47 | 3.85 | 2.48 | 8.80 |
| 10 | Dauk-yat | Myrtaceae | *Tristaniopsis burmanica* (Griff.) P.G. Wilson & J.T. Waterh. | 3.93 | 3.85 | 0.97 | 8.74 |
|  | Others |  |  | 45.27 | 62.09 | 45.41 | 152.77 |
|  | **Total** |  |  | **100.00** | **100.00** | **100.00** | **300.00** |

(d) Monasteries (Small trees with DBH<5 cm, H>1.3 m) **(Shannon diversity index H′ = 3.17)**

| **No.** | **Local name** | **Family name** | **Species name** | **Relative density** | **Relative frequency** | **Relative dominance** | **IVI** |
| --- | --- | --- | --- | --- | --- | --- | --- |
| 1 | Thaik-wa | Poaceae | *Bambusa tulda* Roxb. | 8.24 | 4.35 | 11.37 | 23.95 |
| 2 | Taw-thi-din | Euphorbiaceae | *Mallotus philippinensis* (Lam.) Muell. Arg. | 7.06 | 6.52 | 8.93 | 22.51 |
| 3 | Thitni | Rubiaceae | *Wendlandia tinctoria* DC. | 7.06 | 2.17 | 10.66 | 19.89 |
| 4 | Kywet-sa-ni | Fagaceae | *Lithocarpus dealbatus* (Hook. f. & Thoms.) | 8.24 | 4.35 | 6.93 | 19.52 |
| 5 | Dauk-yat | Myrtaceae | *Tristaniopsis burmanica* (Griff.) P.G. Wilson & J.T. Waterh. | 5.88 | 6.52 | 5.51 | 17.92 |
| 6 | Taung-zalat | Apocynaceae | *Rauvolfia microcarpa* Hook. f. | 5.88 | 6.52 | 4.57 | 16.97 |
| 7 | Thetyin-gyi | Euphorbiaceae | *Croton oblongifolius* Roxb. | 5.88 | 6.52 | 4.22 | 16.62 |
| 8 | Tabyae-phyu | Myrtaceae | *Syzygium cumini* (L.) Skeels | 5.88 | 4.35 | 4.51 | 14.74 |
| 9 | Thite | Fagaceae | *Quercus mespilifolia* Wall. | 3.53 | 4.35 | 6.44 | 14.32 |
| 10 | Win-u | Fabaceae | *Millettia eriocalyx* Dunn | 5.88 | 2.17 | 4.24 | 12.29 |
|  | Others |  |  | 36.47 | 52.17 | 32.61 | 121.26 |
|  | **Total** |  |  | **100.00** | **100.00** | **100.00** | **300.00** |

(e) Golf course (Large trees with DBH≥5 cm, H>1.3 m) **(Shannon diversity index H′ = 1.57)**

| **No.** | **Local name** | **Family name** | **Species name** | **Relative density** | **Relative frequency** | **Relative dominance** | **IVI** |
| --- | --- | --- | --- | --- | --- | --- | --- |
| 1 | Acacia | Fabaceae | *Acacia auriculiformis* A.Cunn.ex Benth. | 5.66 | 8.33 | 3.89 | 17.88 |
| 2 | Dauk-yat | Myrtaceae | *Tristaniopsis burmanica* (Griff.) P.G. Wilson & J.T. Waterh. | 1.89 | 8.33 | 0.79 | 11.01 |
| 3 | Htinyu | Pinaceae | *Pinus insularis* Endl. | 52.83 | 16.67 | 72.06 | 141.56 |
| 4 | Metkauk | Dilleniaceae | *Dillenia aurea* Sm. | 1.89 | 8.33 | 0.33 | 10.55 |
| 5 | Payok | Lauraceae | *Cinnamomum camphora* (L.) Nees & Eberm. | 5.66 | 8.33 | 1.19 | 15.18 |
| 6 | Taung-tama | Meliaceae | *Cedrela serrata* Royle | 1.89 | 8.33 | 0.07 | 10.29 |
| 7 | Thayet (Mango) | Anacardiaceae | *Mangifera indica* L. | 1.89 | 8.33 | 0.28 | 10.50 |
| 8 | Thit-e | Fagaceae | *Quercus serrata* Thunb. | 15.09 | 8.33 | 15.75 | 39.18 |
| 9 | Thityah | Theaceae | *Schima wallichii* (DC.) Korth. | 9.43 | 16.67 | 5.09 | 31.19 |
| 10 | U-byat | Ericaceae | *Craibiodendron stellatum* W.W. Sm. | 3.77 | 8.33 | 0.53 | 12.63 |
|  | **Total** |  |  | **100.00** | **100.00** | **100.00** | **300.00** |

(f) Coffee farms (Large trees with DBH≥5 cm, H>1.3 m) **(Shannon diversity index H′ = 1.14)**

| **No.** | **Local name** | **Family name** | **Species name** | **Relative density** | **Relative frequency** | **Relative dominance** | **IVI** |
| --- | --- | --- | --- | --- | --- | --- | --- |
| 1 | Silver oak | Proteaceae | *Grevillea robusta* A. Cunn. ex R. Br. | 70.23 | 42.11 | 80.37 | 192.71 |
| 2 | Magadamea | Proteaceae | *Macadamia ternifolia* F.Muell. | 12.98 | 10.53 | 1.32 | 24.83 |
| 3 | Ye-badon | Euphorbiaceae | *Sapium baccatum* Roxb. | 3.82 | 5.26 | 11.70 | 20.78 |
| 4 | Pyin-oo-hwin | Cupressaceae | *Cupressus goveniana* var. knightiana Rehd. | 2.29 | 10.53 | 1.62 | 14.44 |
| 5 | (Peinne)Jackfruit | Moraceae | *Artocarpus heterophyllus* Lam. | 3.82 | 5.26 | 1.97 | 11.05 |
| 6 | Kathit | Fabaceae | *Erythrina variegata* L. | 2.29 | 5.26 | 1.96 | 9.51 |
| 7 | Thayet (Mango) | Anacardiaceae | *Mangifera indica* L. | 1.53 | 5.26 | 0.56 | 7.35 |
| 8 | Ondon | Lauraceae | *Litsea glutinosa* (Lour.) C.B.Rob. | 1.53 | 5.26 | 0.26 | 7.05 |
| 9 | Taw-thidin | Euphorbiaceae | *Mallotus philippinensis* (Lam.) Muell. Arg. | 0.76 | 5.26 | 0.16 | 6.18 |
| 10 | Htawbat(Avocado) | Lauraceae | *Persea americana* Mill. | 0.76 | 5.26 | 0.08 | 6.11 |
|  | **Total** |  |  | **100.00** | **100.00** | **100.00** | **300.00** |

(g) Coffee farms (Small trees with DBH<5 cm, H>1.3 m) **(Shannon diversity index H′ = 0.26)**

| **No.** | **Local name** | **Family name** | **Species name** | **Relative density** | **Relative frequency** | **Relative dominance** | **IVI** |
| --- | --- | --- | --- | --- | --- | --- | --- |
| 1 | Coffee | Rubiaceae | *Coffea arabica* L. | 94.79 | 66.67 | 92.96 | 254.42 |
| 2 | Silver oak | Proteaceae | *Grevillea robusta* A. Cunn. ex R. Br. | 0.27 | 8.33 | 0.38 | 8.99 |
| 3 | Met mann | Rosaceae | *Prunus communis* Huds. | 3.01 | 8.33 | 1.56 | 12.91 |
| 4 | Thayet (Mango) | Anacardiaceae | *Mangifera indica* L. | 1.10 | 8.33 | 4.21 | 13.64 |
| 5 | Ka thit | Fabaceae | *Erythrina variegata* L. | 0.82 | 8.33 | 0.85 | 10.01 |
|  | **Total** |  |  | **100.00** | **100.00** | **100.00** | **300.00** |
