# Supplementary material for: Land use change, carbon stocks and tree species diversity in green spaces of a secondary city in Myanmar, Pyin Oo Lwin
Source: PLoS One. 2019 Nov 26;14(11):e0225331. doi: 10.1371/journal.pone.0225331 (PMC6879162; doi:10.1371/journal.pone.0225331)
Supplement: S6 Table — (DOCX) [file pone.0225331.s009.docx]

S6 Table: Carbon stocks component and Shannon diversity index for each plot

| **Urban green** | **Plot no.** | **Above ground carbon stocks (t/ha)** | **Belowground carbon stocks (t/ha)** | **Soil organic carbon stocks (t/ha)** | **Shannon diversity index** |
| --- | --- | --- | --- | --- | --- |
| Botanical garden | 1 | 76.47 | 17.67 | 232.29 | 0.36 |
|  | 2 | 230.21 | 54.99 | 266.92 | 0.36 |
|  | 3 | 452.00 | 108.23 | 194.18 | 0.36 |
|  | 4 | 154.92 | 36.89 | 177.83 | 0.32 |
|  | 5 | 0.00 | 0.00 | 218.20 | 0.00 |
|  | 6 | 0.00 | 0.00 | 153.47 | 0.00 |
|  | 7 | 159.21 | 38.01 | 266.22 | 0.36 |
|  | 8 | 96.28 | 24.44 | 207.06 | 0.26 |
|  | 9 | 128.74 | 30.44 | 178.70 | 0.32 |
|  | 10 | 131.13 | 31.01 | 171.22 | 0.36 |
| Monasteries | 1 | 130.53 | 31.22 | 241.16 | 0.28 |
|  | 2 | 105.01 | 25.14 | 172.26 | 0.34 |
|  | 3 | 47.51 | 11.33 | 144.07 | 0.36 |
|  | 4 | 49.10 | 11.72 | 177.48 | 0.30 |
|  | 5 | 100.04 | 23.86 | 154.86 | 0.36 |
|  | 6 | 90.11 | 42.50 | 219.24 | 0.35 |
|  | 7 | 89.79 | 21.47 | 182.70 | 0.21 |
|  | 8 | 137.40 | 32.83 | 192.27 | 0.36 |
|  | 9 | 93.88 | 22.35 | 170.35 | 0.37 |
|  | 10 | 196.40 | 47.04 | 53.59 | 0.37 |
|  | 11 | 19.36 | 4.55 | 116.93 | 0.36 |
|  | 12 | 68.08 | 16.04 | 158.17 | 0.36 |
|  | 13 | 373.21 | 89.29 | 132.76 | 0.37 |
|  | 14 | 38.44 | 9.05 | 128.41 | 0.36 |
|  | 15 | 32.17 | 7.66 | 99.18 | 0.28 |
|  | 16 | 79.17 | 18.91 | 105.97 | 0.32 |
|  | 17 | 14.17 | 3.18 | 179.57 | 0.36 |
| Coffee farms | 1 | 142.64 | 34.17 | 153.47 | 0.23 |
|  | 2 | 54.40 | 13.01 | 89.78 | 0.19 |
|  | 3 | 156.09 | 37.35 | 123.89 | 0.24 |
|  | 4 | 63.54 | 14.88 | 160.78 | 0.25 |
|  | 5 | 215.36 | 51.37 | 202.54 | 0.13 |
|  | 6 | 315.29 | 75.53 | 130.33 | 0.10 |
|  | 7 | 316.36 | 75.94 | 81.43 | 0.18 |
|  | 8 | 192.19 | 45.96 | 98.83 | 0.13 |
| Golf course | 1 | 0.00 | 0.00 | 114.84 | 0.00 |
|  | 2 | 89.46 | 21.42 | 194.88 | 0.36 |
|  | 3 | 3.42 | 0.00 | 106.14 | 0.00 |
|  | 4 | 0.00 | 0.00 | 77.95 | 0.00 |
|  | 5 | 60.79 | 14.21 | 148.25 | 0.36 |
|  | 6 | 195.73 | 46.66 | 176.96 | 0.21 |
| Seasonal farms | 1 | 5.00 | 1.20 | 173.83 | 0.00 |
|  | 2 | 5.00 | 1.20 | 115.88 | 0.00 |
|  | 3 | 5.00 | 1.20 | 36.19 | 0.00 |
|  | 4 | 5.00 | 1.20 | 66.29 | 0.00 |
|  | 5 | 5.00 | 1.20 | 117.97 | 0.00 |
|  | 6 | 5.00 | 1.20 | 174.00 | 0.00 |
|  | 7 | 5.00 | 1.20 | 174.00 | 0.00 |
|  | 8 | 5.00 | 1.20 | 145.64 | 0.00 |
|  | 9 | 5.00 | 1.20 | 119.71 | 0.00 |
|  | 10 | 5.00 | 1.20 | 98.83 | 0.00 |
|  | 11 | 5.00 | 1.20 | 45.94 | 0.00 |
|  | 12 | 5.00 | 1.20 | 173.83 | 0.00 |
|  | 13 | 5.00 | 1.20 | 171.74 | 0.00 |
|  | 14 | 5.00 | 1.20 | 97.44 | 0.00 |
|  | 15 | 5.00 | 1.20 | 119.02 | 0.00 |
|  | 16 | 5.00 | 1.20 | 142.51 | 0.00 |
|  | 17 | 5.00 | 1.20 | 93.79 | 0.00 |
|  | 18 | 5.00 | 1.20 | 126.32 | 0.00 |
|  | 19 | 5.00 | 1.20 | 89.78 | 0.00 |
|  | 20 | 5.00 | 1.20 | 98.83 | 0.00 |
|  | 21 | 5.00 | 1.20 | 116.93 | 0.00 |
|  | 22 | 5.00 | 1.20 | 149.29 | 0.00 |
|  | 23 | 5.00 | 1.20 | 58.12 | 0.00 |
|  | 24 | 5.00 | 1.20 | 118.32 | 0.00 |
|  | 25 | 5.00 | 1.20 | 101.27 | 0.00 |
