# Supplementary material for: Land use change, carbon stocks and tree species diversity in green spaces of a secondary city in Myanmar, Pyin Oo Lwin
Source: PLoS One. 2019 Nov 26;14(11):e0225331. doi: 10.1371/journal.pone.0225331 (PMC6879162; doi:10.1371/journal.pone.0225331)
Supplement: S7 Table — (DOCX) [file pone.0225331.s010.docx]

S7 Table. Carbon stock for each green space

| **No.** | **Fig. no.** | **Area (ha)** | **Uses** | **Total carbon (t/ha)** | **Total carbon per green space (t)** |
| --- | --- | --- | --- | --- | --- |
| 1. | 1 | 177.00 | Botanical garden | 383.67 | 67909.59 |
| 2. | 2a | 5.68 | Monastery | 286.63 | 1628.06 |
| 3. | 2b | 1.83 | Monastery | 278.76 | 510.13 |
| 4. | 2c | 7.14 | Monastery | 323.72 | 2311.36 |
| 5. | 2d | 1.33 | Monastery | 218.94 | 291.19 |
| 6. | 2e | 4.27 | Monastery | 418.77 | 1788.15 |
| 7. | 2f | 20.82 | Monastery | 178.97 | 3726.16 |
| 8. | 3a | 132.05 | Coffee farm | 268.26 | 35423.73 |
| 9. | 3b | 86.30 | Coffee farm | 409.87 | 35371.78 |
| 10 | 3c | 23.30 | Coffee farm | 473.73 | 11037.91 |
| 11. | 3d | 3.07 | Coffee farm | 336.98 | 1034.53 |
| 12 | 4 | 104.59 | Golf course | 208.45 | 21801.79 |
| 13 | 5a | 1.50 | Seasonal farm | 119.39 | 179.09 |
| 14 | 5b | 0.97 | Seasonal farm | 112.95 | 109.56 |
| 15 | 5c | 1.42 | Seasonal farm | 114.17 | 162.12 |
| 16 | 5d | 0.57 | Seasonal farm | 128.78 | 73.40 |
| 17 | 5e | 1.25 | Seasonal farm | 108.23 | 135.29 |
| 18 | 5f | 2.12 | Seasonal farm | 159.54 | 338.22 |
