# Supplementary material for: Land use change, carbon stocks and tree species diversity in green spaces of a secondary city in Myanmar, Pyin Oo Lwin
Source: PLoS One. 2019 Nov 26;14(11):e0225331. doi: 10.1371/journal.pone.0225331 (PMC6879162; doi:10.1371/journal.pone.0225331)
Supplement: S8 Table — (DOCX) [file pone.0225331.s011.docx]

S8 Table. Summary statistics for carbon stock components

| **Variable** | **Observation** | **Mean** | **Std. Dev.** | **Min** | **Max** |
| --- | --- | --- | --- | --- | --- |
| Aboveground carbon | 66 | 76.12 | 98.98 | 0 | 452.00 |
| Belowground carbon | 66 | 18.49 | 23.89 | 0 | 108.23 |
| Soil organic carbon | 66 | 143.65 | 51.06 | 36.19 | 266.92 |
| Total carbon | 66 | 238.25 | 145.96 | 42.39 | 754.42 |
