# Supplementary material for: Land use change, carbon stocks and tree species diversity in green spaces of a secondary city in Myanmar, Pyin Oo Lwin
Source: PLoS One. 2019 Nov 26;14(11):e0225331. doi: 10.1371/journal.pone.0225331 (PMC6879162; doi:10.1371/journal.pone.0225331)
Supplement: S9 Table — (DOCX) [file pone.0225331.s012.docx]

Table S9: Kruskal-Wallis test for carbon stock components

| **Variable** | **Chi-square with ties (X^2^)** | **p-value** |
| --- | --- | --- |
| Aboveground carbon | 54.73 | 0.0001 |
| Belowground carbon | 54.66 | 0.0001 |
| Soil organic carbon | 17.89 | 0.0005 |
| Total carbon | 42.61 | 0.0001 |
